# A modular pathway engineering strategy for the high-level production of β-ionone in *Yarrowia lipolytica*

**DOI:** 10.1186/s12934-020-01309-0

**Published:** 2020-02-27

**Authors:** Yanping Lu, Qingyu Yang, Zhanglin Lin, Xiaofeng Yang

**Affiliations:** School of Biology and Biological Engineering, South China University of Technology, 382 East Outer Loop Road, University Park, Guangzhou, 510006 China

**Keywords:** Metabolic engineering, β-ionone, PK–PTA pathway, Acetyl-CoA, Fermentation optimization, Dissolved oxygen, *Yarrowia lipolytica*

## Abstract

**Background:**

The GRAS and oleaginous yeast *Yarrowia lipolytica* (*Y. lipolytica*) is an attractive cell factory for the production of chemicals and biofuels. The production of many natural products of commercial interest have been investigated in this cell factory by introducing heterologous biosynthetic pathways and by modifying the endogenous pathways. However, since natural products anabolism involves long pathways and complex regulation, re-channelling carbon into the product of target compounds is still a cumbersome work, and often resulting in low production performance.

**Results:**

In this work, the carotenogenic genes contained *carB* and bi-functional *carRP* from *Mucor circinelloides* and carotenoid cleavage dioxygenase 1 (*CCD1*) from *Petunia hybrida* were introduced to *Y. lipolytica* and led to the low production of β-ionone of 3.5 mg/L. To further improve the β-ionone synthesis, we implemented a modular engineering strategy for the construction and optimization of a biosynthetic pathway for the overproduction of β-ionone in *Y. lipolytica*. The strategy involved the enhancement of the cytosolic acetyl-CoA supply and the increase of MVA pathway flux, yielding a β-ionone titer of 358 mg/L in shake-flask fermentation and approximately 1 g/L (~ 280-fold higher than the baseline strain) in fed-batch fermentation.

**Conclusions:**

An efficient β-ionone producing GRAS *Y. lipolytica* platform was constructed by combining integrated overexpressed of heterologous and native genes. A modular engineering strategy involved the optimization pathway and fermentation condition was investigated in the engineered strain and the highest β-ionone titer reported to date by a cell factory was achieved. This effective strategy can be adapted to enhance the biosynthesis of other terpenoids in *Y. lipolytica*.

## Background

Terpenoids and their derivatives have attracted great interest for their commercial application as biofuels, flavoring ingredients, fragrances, antiseptics and pharmaceuticals [1–4]. β-ionone, an apocarotenoid derived from carotenoids (C40 terpenoids), has a warm, woody, and violet-like aroma, a low odor threshold, and has been widely used in the food and cosmetic industries [5]. Moreover, β-ionone is a key intermediate for the synthesis of vitamins A, E and K [6]. Currently, the annual production of β-ionone is about 4000–8000 tonnes and the demand is rapidly increasing [1]. Due to the long grow cycle and low concentration in plants, the direct extraction of β-ionone from plants affords low yields of the terpenoid, insufficient to meet the demand [4, 7]. Commercial β-ionone is mainly produced by chemical synthesis, which is characterized by the formation of undesirable byproducts and is not environmentally friendly [8]. These concerns have stimulated increased efforts to develop microbial cell factories for the production of natural products from carbohydrate feedstocks. The use of generally recognized as safe (GRAS) microorganisms to convert natural raw materials into products leads to aroma compounds that can be described as natural, if these compounds are known to be components of the natural raw materials, according to the United States and the European Union flavor regulations [9]. As a consequence, companies and researchers have shifted their focus from *Escherichia coli* (*E. coli*) to GRAS microorganisms, such as yeasts, for terpenoids production [10–13].

Recently, the GRAS, unconventional yeast *Y. lipolytica* has rapidly emerged as a valuable cell factory for the production of terpenoids due to its own endogenous mevalonate pathway (MVA) and its high lipid production capacity [7, 12]. Metabolic engineering and synthetic biology offer the ability of rewiring microbial carbon fluxes to create efficient cell factories for the production of natural products. To date, this yeast has been successfully engineered for the production of monoterpenoids, e.g. 23.6 mg/L limonene [14] and 7.0 mg/L linalool [15], sesquiterpenoids, e.g. 978.2 μg/L (+)-nootkatone [16], and tetraterpenoids, e.g. 242.0 mg/L lycopene [17], 6.5 g/L β-carotene [18] and 54.6 mg/L astaxanthin [19].

In microbial cell factories engineered for commercial production, pathway design and optimization are essential steps [20, 21]. Ajikumar et al. [22] established a multivariate modular metabolic engineering (MMME) approach, which segments metabolic pathways into modules regulated by distinct promoters, to enhance taxadiene (a taxol intermediate) production by ~ 15,000 fold in *E. coli*. Variations of this modularization strategy have been implemented in *E. coli* [23, 24] and in *S. cerevisiae* [25, 26].

Additionally, efficient and predictable synthetic biology tools have been developed that facilitate the metabolic engineering of *Y. lipolytica*. Popular approaches for assembling the different DNA parts into gene cassettes, such as Gibson assembly, Gateway and Golden Gate strategies, have also been applied to the engineering of *Y. lipolytica* [18, 27]. These gene cassettes can be further simplified and efficiently edited by the powerful tool CRISPR–Cas9, which was implemented at 2016 [28]. The CRISPR interference (CRISPRi) system was also established for gene expression modification in *Y. lipolytica* [29].

**Fig. 1:**
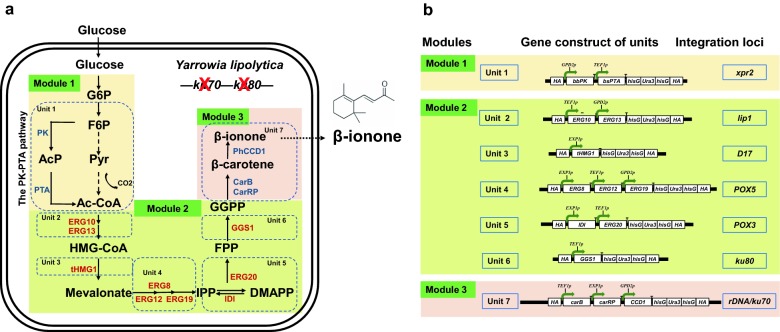
β-ionone biosynthesis pathway in the engineered *Yarrowia lipolytica*. **a** The biosynthesis pathway was organized into three modules: Module 1, the cytosolic acetyl-CoA supply module; Module 2, the MVA module; Module 3, the β-ionone synthesis module. Exogenous and endogenous genes are indicated in blue and red, respectively. **b** The detailed view of all the seven units. The genes expressed encode the following enzymes: *PK* phosphoketolase, *PTA* phosphotransacetylase, *ERG10* acetoacetyl-CoA thiolase, *ERG13* hydroxymethylglutaryl-CoA synthase, *tHMGR* truncated hydroxymethylglutaryl-CoA reductase, *ERG8* phosphomevalonate kinase, *ERG12* mevalonate kinase, *ERG19* mevalonate diphosphate decarboxylase, *IDI* isopentenyl diphosphate isomerase, *ERG20* geranyl/farnesyl diphosphate synthase, *GGS1* GGPP synthase, *carB* phytoene dehydrogenase, *carRP* phytoene synthase/lycopene cyclase, *CCD1* carotenoid cleavage dioxygenase

In this study, we used a modular pathway engineering approach for the construction and optimization of a long metabolic pathway, for the overproduction of β-ionone in *Y. lipolytica*. The whole pathway was divided into three modules, which were then segmented into a total of seven units for genome integration (Fig. 1). These units and modules were designed to enable quick assembly, integration and step-wise evaluation of the β-ionone synthesis pathway in *Y. lipolytica*. The best performing strain yielded 358 mg/L β-ionone accumulation in shake-flask fermentation and 0.98 g/L in fed-batch fermentation using a 3-L bioreactor, the highest titers for β-ionone obtained in microbial cell factories reported to date. During the course of this work, Czajka et al. [30] engineered *Y. lipolytica* to produce 68 mg/L in flask fermentation and 380 mg/L in a 2-L bioreactor by the integrated overexpression of the MVA and β-ionone synthesis pathway genes, and fermentation optimization. In this work, we applied a different modular integration approach that allowed for evaluation of different units of the genes along the whole pathway, and used the CRISPR–Cas9 technique, which is more convenient than the Cre-loxP technique [30]. In particular, we introduced a new factor, the acetyl-CoA supply module, to increase the availability of acetyl-CoA in the cytosol. We also found that the dissolved oxygen level was a critical parameter for β-ionone biosynthesis. This work should advance the potential of *Y. lipolytica* as a cell factory platform for the high-level production of terpenoids.

## Results

### Re-casting the β-ionone biosynthesis into three modules and the choice of *Y. lipolytica* strain

The overall pathway from glucose to β-ionone was organized into the following three modules, considering the two most important intermediates: acetyl-CoA and geranylgeranyl diphosphate (GGPP) [31–33] (Fig. 1a, b). (1) Module 1 (the cytosolic acetyl-CoA supply module): from glucose to acetyl-CoA; in this module, the metabolic flux from F6P to acetyl-CoA was rewired by the introduction of the two genes of the PK–PTA pathway [34]. (2) Module 2 (the MVA module): from acetyl-CoA to GGPP. (3) Module 3 (the β-ionone synthesis module): from GGPP to β-ionone. These modules were further divided into seven units for integration with DNA sizes ranging from 7.5 to 14.5 kb (Table 1). For the *Y. lipolytica* strain, we knocked out the *ku70* and *ku80* genes of *Y. lipolytica* Po1f, to generate strain YLBI0003, in order to improve the homologous recombination (HR) efficiency, as previously reported [28, 35, 36] (Additional file 1: Fig. S1). Three modules were then successively integrated into strain YLBI0003 by reiterative recombination via the single plasmid CRISPR–Cas9-mediated genome editing system based on pCAS1yl [28], in the following order: modules 3, 2, 1.

**Table 1:** The modules and units constructed in this work

Module	Unit	Name	Length/bp	Locus (first copy)	Locus (multi copy)
1	1	bbPK-bsPTA	11,234	*rDNA*	*pox4*, *xpr2*
2	2	ERG10-ERG13	11,451	*lip1*	
	3	tHMG1	8820	*D17*	
	4	ERG8-ERG12-ERG19	13,425	*pox5*	
	5	IDI-ERG20	10,044	*pox3*	
	6	GGS1	7527	*ku80*	
3	7	carB-carRP-CCD1	14,440	*rDNA*	*ku70*

### Enhancing the MVA module

The β-ionone synthesis module (Module 3) was first inserted into the *rDNA* locus of strain YLBI0003, the generated strain was designated as the baseline strain YLBI0004. In this module, the genes *carB*, *carRP* and *CCD1* were placed under the strong constitutive promoters *P*_*TEF1*_, *P*_*EXP1*_, and *P*_*GPD2*_, respectively. This strain only yielded 3.5 ± 0.2 mg/L β-ionone when cultured in YPD medium at 20 °C for 12 days (Additional file 1: Fig. S2a). Although the strain yielded a slightly higher β-ionone titer when the fermentation temperature was set at 15 °C, the higher cost of maintaining cultures at low temperature made us opt for 20 °C as the fermentation temperature during the optimization steps (Additional file 1: Fig. S2b).

**Fig. 2:**
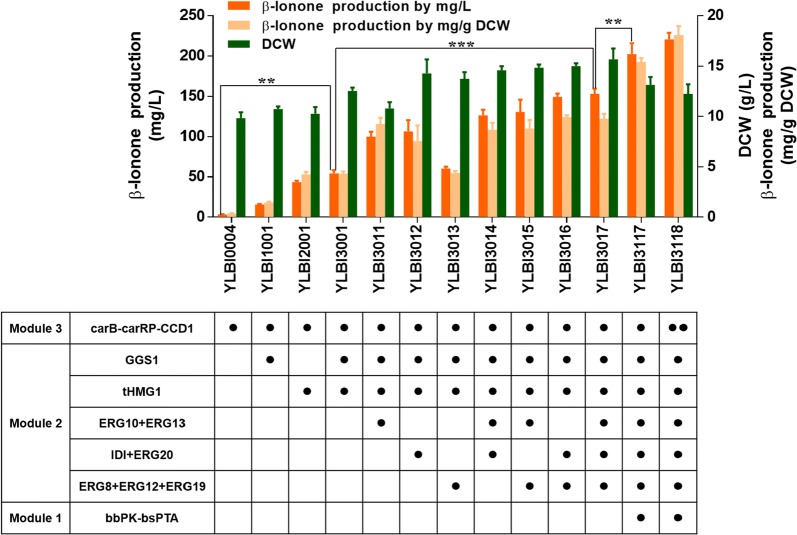
β-ionone production in different engineered strains. Data represent the mean ± standard deviation (n = 3). Significance was marked by t-test, **p-value ≤ 0.01; ***p-value ≤ 0.001

Module 2, which controls the transformation of acetyl-CoA to GGPP, contains nine genes. Among them, two key genes hydroxymethylglutaryl-CoA reductase (*HMGR1*) and geranylgeranyl diphosphate synthase (*GGS1*) were singularly overexpressed or co-overexpressed in strain YLBI0004 under the control of *P*_*EXP1*_ and *P*_*TEF1*_, respectively [36, 37]. For *HMGR1*, we used a truncated variant (*tHMGR1*) lacking the first 500 amino acid residues at the N-terminus, which was found to be more stable in the cytoplasm [37]. The strain YLBI3001, which overexpressed both *tHMG1* and *GGS1* genes, produced more β-ionone than the strains overexpressing only one of them (Fig. 2). This strain accumulated 54.2 ± 4.0 mg/L (4.3 ± 0.2 mg/g DCW) β-ionone, representing a 15-fold increase compared to the baseline strain YLBI0004.

To further push the carbon flux from acetyl-CoA to β-ionone, the other three units containing 7 genes, *ERG10* (YALI0E11099g), *ERG13* (YALI0F30481g), *ERG8* (YALI0E06193g), *ERG12* (YALI0B16038g), *ERG19* (YALI0F05632g), *IDI* (YALI0F04015g), and *ERG20* (YALI0E05753g) were sequentially integrated downstream of native constitutive strong promoters (*P*_*TEF1*_, *P*_*EXP1*_ or *P*_*GPD2*_) into strain YLBI3001 (Fig. 1). Different integration combinations of the three units were investigated, and the strain harboring all three units (YLBI3017) showed the highest β-ionone titer of 152.9 ± 5.8 mg/L (9.8 ± 0.5 mg/g DCW). Therefore, overexpression of the whole Module 2 achieved a 43.7-fold increase in β-ionone yield compared to the baseline strain YLBI0004, or a 2.8-fold increase over the strain YLBI3001.

### Enhancing the cytosolic acetyl-CoA supply by introduction of the PK–PTA pathway genes

In strain YLBI3017, the genes of Module 2 and 3 were overexpressed in cytoplasm, while the precursor acetyl-CoA was generated in the mitochondria, shuttled to the cytoplasm as citrate, and then converted to acetyl-CoA by acetyl-CoA synthetase. By this native pathway of cytosolic acetyl-CoA generation from glucose, there are as much as 14 steps that only a maximum of 2 mol of acetyl-CoA can be produced from 1 mol of glucose with 2 mol of carbon loss. The recently reported PK–PTA pathway, a non-oxidative glycolytic pathway which consists of phosphoketolase (PK) and phosphotransacetylase (PTA), can yield a maximum of 3 mol of acetyl-CoA directly in the cytoplasm from 1 mol of glucose, with only four steps [34]. This pathway was successfully introduced into *Y. lipolytica* to yield a 16.4% increase of lipid titer [38].

**Fig. 3:**
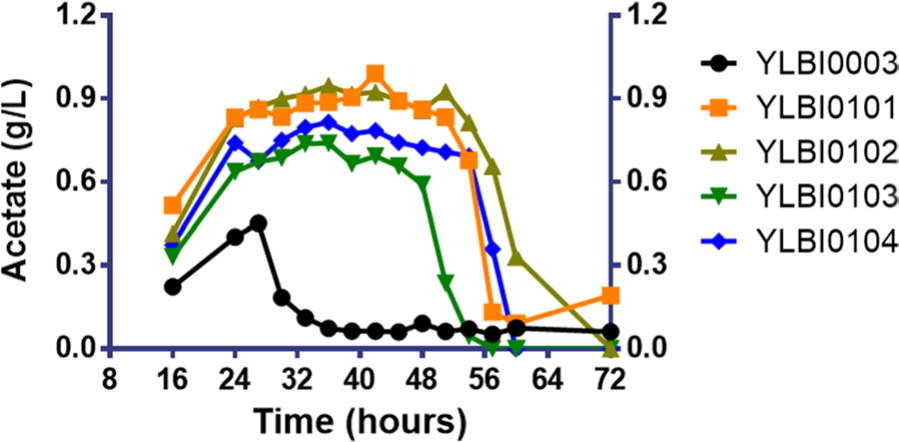
PK–PTA pathway correlated with acetate accumulation. Acetate accumulation of all four combinations during the whole growth phase. *Y. lipolytica* po1f was using as native control. *lmPK* from *Leuconostoc mesenteroides*, *bbPK* from *Bifidobacterium bifidum*, *ckPTA* from *Clostridium kluyveri*, *bsPTA* from *Bacillus subtilis*. Data represent the mean ± standard deviation (n = 3)

In this work, two heterologous PKs under the control of *P*_*GPD2*_ (*lmPK* from *Leuconostoc mesenteroides*; *bbPK* from *Bifidobacterium bifidum*) and two heterologous PTAs under the control of *P*_*TEF1*_ (*ckPTA* from *Clostridium kluyveri*; *bsPTA* from *Bacillus subtilis*) were assembled resulting in four combinations, and were first investigated separately using the baseline strain YLBI0003. As a metabolic intermediate, acetyl-CoA is rapidly depleted in the cytoplasm, therefore acetate, a derivative of acetyl-CoA, was used as the indicator for the production of acetyl-CoA [34]. The highest acetate titers in the strains containing *bbPK*-*bsPTA*, *bbPK*-*ckPTA*, *lmPK*-*bsPTA* and *lmPK*-*ckPTA* were 0.99 g/L, 0.95 g/L, 0.74 g/L and 0.81 g/L, respectively, representing titers significantly higher than that measured in the baseline strain YLBI0003 (0.45 g/L) (Fig. 3). Based on the hypothesis that the higher acetate production reflected a higher acetyl-CoA supply, *bbPK*-*bsPTA* was selected for further analysis. Moreover, we found that the overexpression of the PK–PTA pathway also enhanced the growth of *Y. lipolytica* (Additional file 1: Fig. S3), in contrast to previous results [34].

The *bbPK*-*bsPTA* was then further evaluated for the effect of different integration loci and copy numbers of PK–PTA pathway, but this time on the basis of strain YLBI3001 that overexpressed *tHMG1* and *GGS1* in addition to Module 3 (Additional file 1: Fig. S4a). The β-ionone titer improved 2.04-fold, 1.93-fold and 2.50-fold (110.3 ± 4.6 mg/L, 104.5 ± 4.5 mg/L and 135.4 ± 3.8 mg/L) when *bbPK*-*bsPTA* was inserted at *rDNA*, *pox4* and *xpr2* loci, respectively (Additional file 1: Fig. S5). However, the increase of *bbPK*-*bsPTA* copy number reduced the titer of β-ionone while promoting the cell growth (Additional file 1: Fig. S5). Thus, subsequently, a copy of *bbPK*-*bsPTA* was integrated at the *xpr2* locus of strain YLBI3017 to generate strain YLBI3117 that harboring all the three modules. The strain YLBI3117 showed a 32% higher β-ionone production than strain YLBI3017 (202.2 ± 13.6 mg/L, 15.4 ± 0.4 mg/g DCW) (Fig. 2).

It was found that 118.8 ± 18.1 mg/L (14.5 ± 3.0 mg/g DCW) β-carotene accumulated in strain YLBI3117 (Additional file 1: Fig. S4b). We thus considered to increase the copy number of Module 3 to improve the production of β-ionone from GGPP. Thus, another copy of Module 3 was integrated at *ku70* locus of strain YLBI3117 to generate strain YLBI3118, but only a slight increase (9%) of β-ionone yield was achieved (Fig. 2). This final strain produced 220.7 ± 8.0 mg/L (18.1 ± 0.9 mg/g DCW) β-ionone in 12 days of shake-flask fermentation while at day 3, day 6 and day 9, the yields were 50.0 ± 3.9 mg/L, 132.1 ± 5.8 mg/L and 154.0 ± 15.7 mg/L, respectively. (Additional file 1: Fig. S6a, b).

Since the final strain YLBI3118 was still *leu* defective, the *leu* gene was then complemented to generate strain YLBI3119. This resulted in an 89% increase in biomass (Additional file 1: Fig. S7). While there was no significant difference in the β-ionone yield (p-value = 0.35) on the basis of volume, however, the yield on the basis of cell mass (mg/g DCW) was significantly reduced from 19.2 ± 2.5 to 10.8 ± 0.5 mg/g DCW.

### High-level production of β-ionone in fed-batch fermentation

**Fig. 4:**
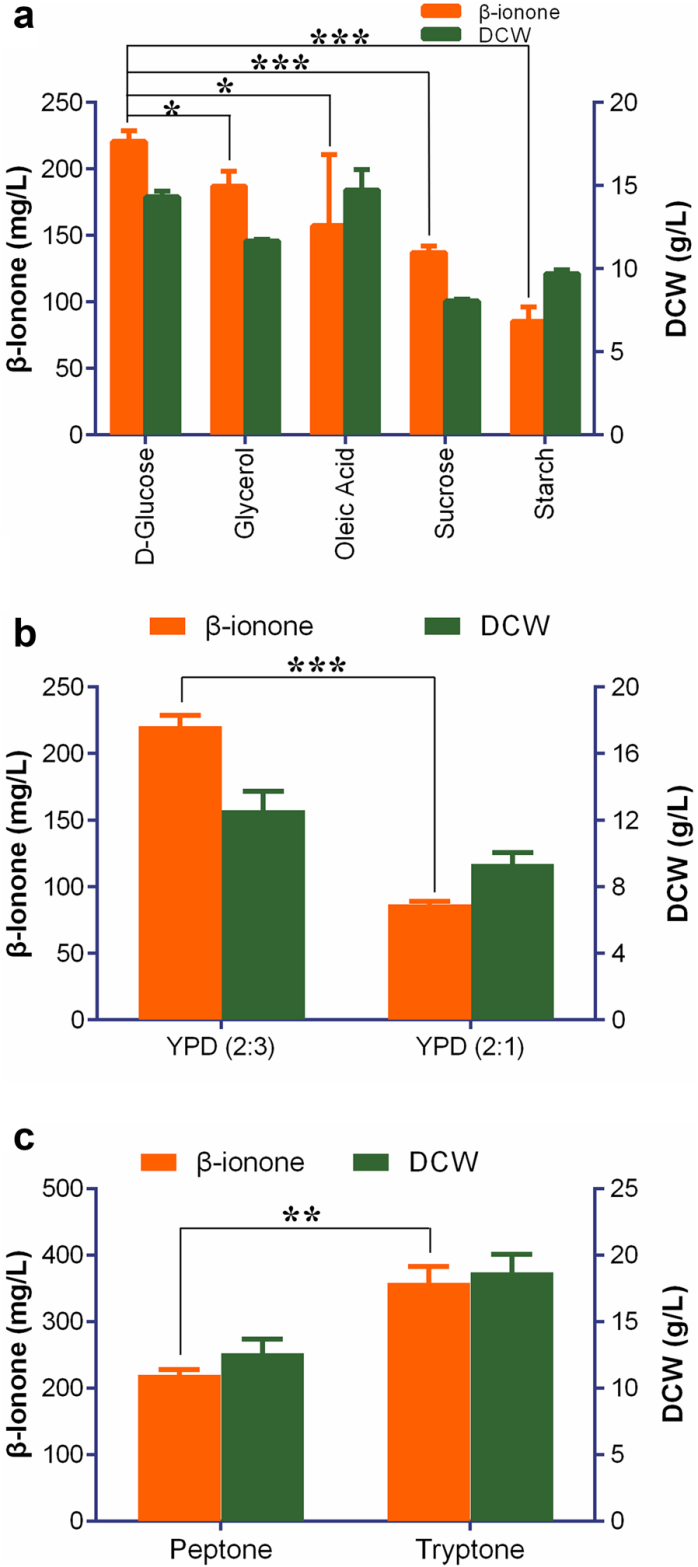
β-ionone flask fermentation optimization. **a** β-ionone production in strain YLBI3118 cultivated in YP media containing 20 g/L of different carbon sources. **b** β-ionone yield in strain YLBI3118 cultured in media containing different carbon/nitrogen ratios. YPDm, modified YPD medium: 3% glucose, 1% peptone, 0.5% yeast extract. **c** β-ionone production in strain YLBI3118 cultured in different nitrogen sources (20 g/L peptone or tryptone) medium. Data represent the mean ± standard deviation (n = 3). Significance was marked by t-test, *p-value ≤ 0.05; **p-value ≤ 0.01; ***p-value ≤ 0.001

Before using the bioreactor fermentation, we investigated the effect of carbon sources and nitrogen sources on the β-ionone production in shake-flasks. 20 g/L of glycerol, starch, sucrose and oleic acid were used to replace glucose for the yeast fermentation, respectively. Although glycerol and oleic acid are commonly regarded as the optimal carbon sources for organic acids or terpenoids production in *Y. lipolytica* [30, 37], we found that glucose was the best carbon source in this case (Fig. 4a). It is likely because for this engineered strain, a maximum of 3 mol of acetyl-CoA could be produced from 1 mol of glucose via the PK–PTA pathway [34] (Fig. 1a), while only a maximum of 1 mol of acetyl-CoA could be produced from 1 mol of glycerol by the glycolysis and the tricarboxylic acid cycle pathways [39]. Moreover, *Y. lipolytica* lacks efficient endogenous enzymes for utilization of sucrose or starch [39, 40]. On the other hand, while 1 mol of oleic acid could yield a maximum of 9 mol of acetyl-CoA, in *Y. lipolytica* oleic acid appears to be utilized more for lipid accumulation [41].

It was reported that nitrogen limitation was favorable for the accumulation of carotenoids [37]. However, we obtained only 86.8 ± 2.5 mg/L (9.3 ± 0.6 mg/g DCW) β-ionone when the amount of nitrogen source (yeast extract and peptone) was reduced according to the carbon to nitrogen (C/N) ratio from 2:3 to 2:1 (wt/wt) (Fig. 4b). This indicates that a rich nitrogen supplement is an important parameter for β-ionone production in strain YLBI3118. Furthermore, the change of peptone with tryptone as the nitrogen source dramatically increased the β-ionone titer up to 358.4 ± 25.0 mg/L (19.2 ± 2.5 mg/g DCW) (Fig. 4c). It should be noted that the β-ionone titer was only calculated from the organic phase, as β-ionone was undetectable in the aqueous medium and was detected only at low concentrations (< 10 mg/L culture) in the cell pellets (data no show).

**Fig. 5:**
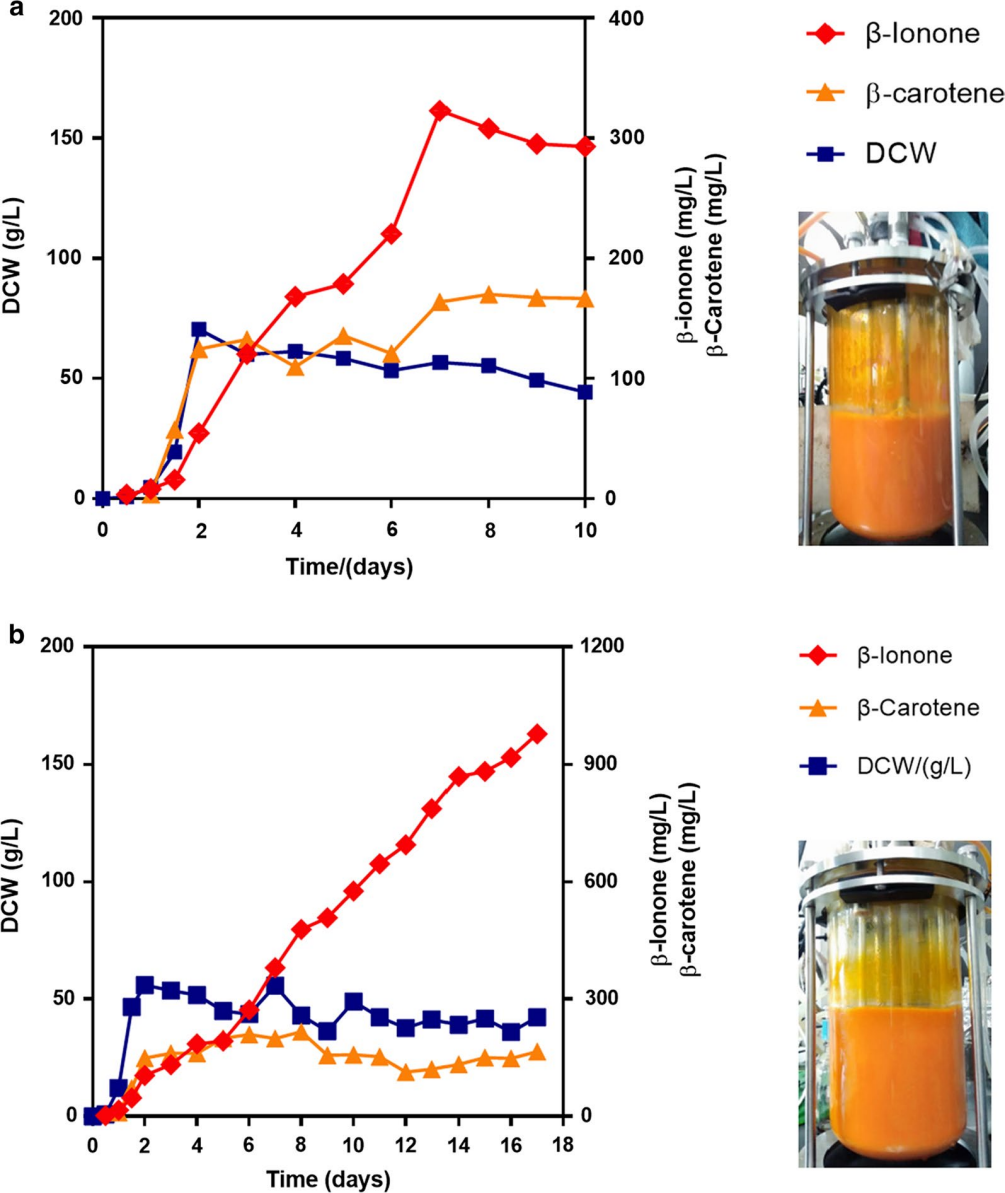
Production of β-ionone by the engineered *Y. lipolytica* in bioreactor by fed-batch fermentation with **a** 5% DO and **b** 15% DO

To further enhance β-ionone production, the fermentation of strain YLBI3118 was performed in 3-L bioreactor. In a first test we observed a long lag phase probably caused by the high-concentration medium used (100 g/L glucose, 50 g/L tryptone, 20 g/L yeast extract), and the titer of β-ionone was only 148 mg/L, much lower than that obtained in shake-flask fermentation (Additional file 1: Fig. S8). In the following test the fermentation was started in 1× YPD medium, then after 12 h of fermentation the medium was fed with 250 mL 10× YPDm supplied at 0.4 mL/min, followed by 600 g/L glucose supplied at 0.1 mL/min. In this batch, the dissolved oxygen (DO) dropped to 0–5% at 12 h till the end of the fermentation. After 7 days (168 h) of fermentation, although the β-ionone productivity increased from 1.2 to 1.9 mg/L/h, the yield resulted 322 mg/L, still lower than that obtained in shake-flask fermentation (Fig. 5a). We speculated that the activity of the dioxygenase CCD1, which converts β-carotene to β-ionone, was limited by the low DO (0–5%). Thus, a series of fed-batch fermentations were conducted by setting the DO to 15%, 25% and 35%. The highest titer of β-ionone (0.98 g/L) was obtained after 17 days (408 h) of fermentation with the DO set at 15% (Fig. 5b). The further increase of DO to 25% and 35% was detrimental to the β-ionone production, and only 0.55 g/L and 0.40 g/L β-ionone were obtained in these conditions, respectively (Additional file 1: Fig. S9a, b). In summary, strain YLBI3118, which was engineered by modular pathway engineering for the overproduction of β-ionone, when cultured the fed-batch fermentation at the optimum DO of 15% in 3-L bioreactor, produced 0.98 g/L β-ionone, which, to the best of our knowledge, is the highest yield of β-ionone obtained in cell factories to date.

## Discussion

A rapidly increasing number of microbial cell factories have been constructed for the biosynthesis of natural products. Two main aspects are important for the application of these cell factories for commercial purposes: the product should be in high yield and regarded as natural. For these reasons, *Y. lipolytica*, a GRAS strain, has attracted great attention in recent years, especially for the production of terpenoids. Several useful synthetic biology tools have been aiding the creation of new and better performing *Y. lipolytica* strains [42, 43]. To date, the highest yields of lipid, lycopene and carotene produced via microbial cell factories have been achieved by using engineered *Y. lipolytica* strains [7, 12].

In this study, a modular pathway engineering method was used to promote the β-ionone production in *Y. lipolytica* (Fig. 1a, Additional file 1: Fig. S10). First, the integration of Module 3 yielded a low concentration of β-ionone. Then, the integration of Module 2 resulted in a 43.7-fold increase of the β-ionone titer. On the basis of this, Module 1 led to a 1.3-fold increase, and another copy of Module 3 led to a 1.1-fold increase, of β-ionone production. Finally, the fermentation optimization achieved a 4.4-fold increase in the titer, or 0.98 g/L of β-ionone. This is significantly higher than the previously reported titer of 380 mg/L [30], although the β-ionone productivity (2.4 mg/L/h) is lower than those of previous studies, e.g. 10 mg/L/h in *E. coli* [23], 2.5 mg/L/h in *S. cerevisiae* [44] and 2.7 mg/L/h in *Y. lipolytica* [30] (Additional file 2: Table S1). Additionally, while our fermentation yields were slightly lower than those obtained in the previous work at similar fermentation intervals [30] (e.g. flask: 50 mg/L at day 3 vs. 68 mg/L at day 4; bioreactor: 272 mg/L at day 6 vs. 380 mg/L at day 6), the β-ionone titer was notably increased to 358 mg/L after 12 days of flask fermentation in the optimized conditions, and 0.98 g/L after 17 days of bioreactor fermentation. This indicates that the extension of fermentation time is favorable for the accumulation of β-ionone.

Within the Module 2, tHMG1 is considered a major rate-limiting enzyme [36]. In our work, the titer of β-ionone increased 13-fold in the strain that overexpressed *tHMG1*. This is consistent with a previous report showing that the β-carotene titer increased 3.4-fold when *tHMG1* was overexpressed [18]. As a future perspective, the integration of multiple copies of *tHMG1* and other MVA related genes could be considered to further increase the flux of MVA pathway [36].

Our study confirmed that the introduction of PK–PTA pathway is a useful route to increase terpenoids production by increasing the production of acetyl-CoA directly in the cytosol. As *Y. lipolytica* is recognized to have a unique propensity for high flux through acetyl-CoA, several groups have focused on using the PK–PTA pathway for the improvement of the synthesis of acetyl-CoA derivatives such as lipid and triacetic acid lactone [4, 30, 32, 45]. To the best of our knowledge, this work is the first that uses the PK–PTA pathway to increase terpenoids production in *Y. lipolytica*.

In the fermentation optimization, we found that a medium rich in nitrogen sources is the optimal choice to achieve the best performance. This result is in sharp contrast to the results obtained for the biosynthesis of β-carotene, which showed an opposite trend [36]. It could be due to that the nitrogen limitation is favorable for the accumulation of lipid bodies and therefore promotes the accumulation of β-carotene which resides in lipid bodies in *Y. lipolytica* [36, 37], while in our case, the β-ionone was transported to the extracellular and enriched in the dodecane. Also, consistent with a previous report in which tryptone was found to improve the production of acetyl-CoA, and to promote the biosynthesis of acetyl-CoA-derived products [46], the β-ionone titer increased 62% when tryptone was used in place of peptone in shake-flask fermentation.

In the subsequent 3-L bioreactor fermentation, it was found that the DO was a very important factor for the biosynthesis of β-ionone. This is consistent with a recent report [47] and is related to the last metabolic step of β-ionone biosynthesis, which is catalyzed by carotenoid cleavage dioxygenase and requires two oxygen molecules to produce two β-ionone molecules. Thus, with the optimization of DO level at 15% the yield of β-ionone increased twofold compared to the bioreactor fermentation performed with DO at 0–5%. As another future perspective, one effective strategy to enhance the biosynthesis of β-ionone could be the introduction of a more powerful CCD1 enzyme to improve the specific cleavage of β-carotene [30]. In another report, protein engineering was performed to improve the membrane affinity of *PhCCD1,* which was derived from *Petunia hybrida*, and 184 mg/L β-ionone was produced in *S. cerevisiae* at the end of 3 days flask fermentation [44]. Thus, the enzyme engineering of CCD1 was an alternative workable method to improve β-ionone biosynthesis.

**Fig. 6:**
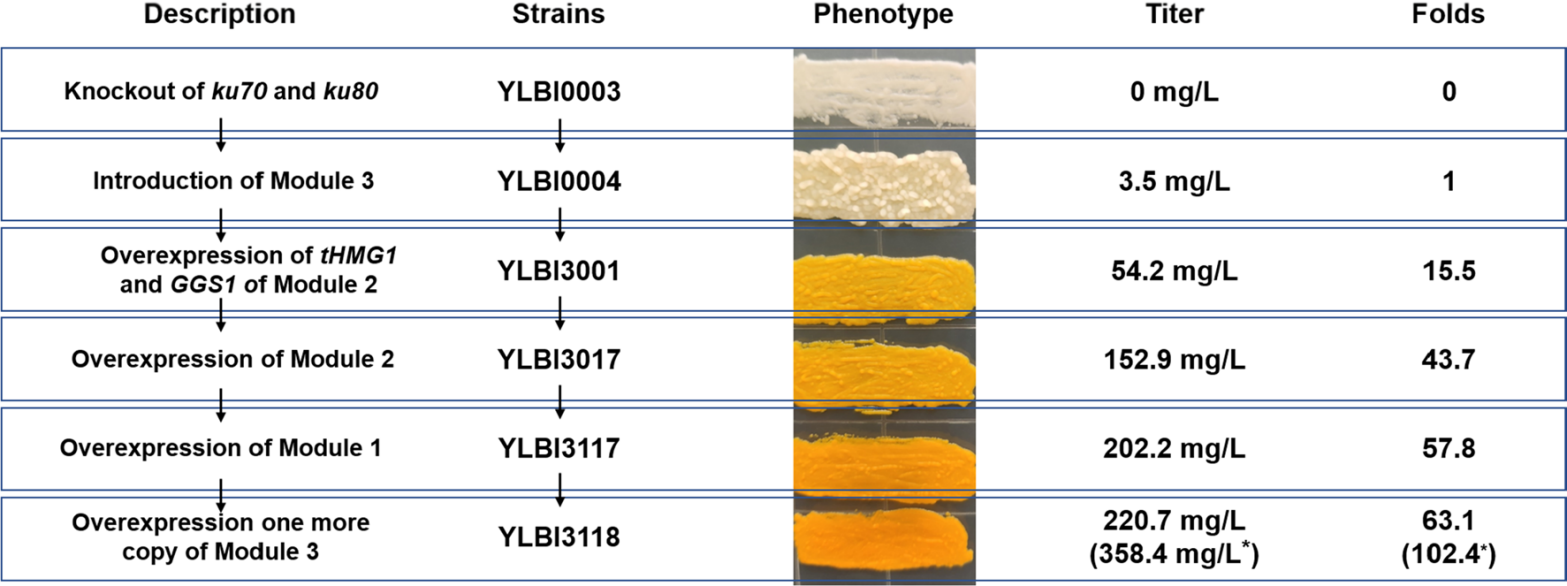
Schematic representation often main steps for the engineering of *Y. lipolytica* strains for β-ionone production. ^*^Titer obtained by culturing on YPD in presence of tryptone instead of peptone as the nitrogen source

Here, *Y. lipolytica* was engineered to become a cell factory and yield the highest β-ionone titer reported to date (Fig. 6). By replacing the relevant genes, the engineered strains could also be adapted for the biosynthesis of many other terpenoids based on the MVA pathway such as taxol [48], artemisinin [49], cannabinoid [50] and many others.

## Conclusions

The GRAS yeast *Y. lipolytica* is an emerging microorganism platform for the production of natural terpenoids and their derivatives. In this work, we used a modular pathway engineering approach based on a set of modules and units for the facile and effective construction of *Y. lipolytica* microbial cell factories for the high-level production of β-ionone. Three modules were responsible for the enhancement of cytosolic acetyl-CoA supply through the PK–PTA pathway, the increase of MVA pathway flux and the synthesis of β-ionone. By the optimization of the design of the three modules, the carbon sources, the nitrogen level and the dissolved oxygen level, the highest titer of 0.98 g/L β-ionone was achieved in a 3-L fermenter by fed-batch fermentation.

## Methods

### Strains, media and culture condition

*Escherichia coli* DH5α was used for cloning and plasmid construction. *E. coli* DH5α was cultured in lysogeny broth (LB, 10 g/L tryptone, 5 g/L yeast extract, and 10 g/L NaCl) supplemented with 50 mg/mL ampicillin at 37 °C under constant shaking. *Y. lipolytica* Po1f (*MatA, leu2*-*270, ura3*-*302, xpr2*-*322, axp1*-*2 Leu*-*, Ura*-*, DAEP, DAXP, Suc*+) was a kind gift from Dr. Catherine Madzak [39]. *Y. lipolytica* strains were cultured in yeast extract peptone dextrose (YPD) medium (10 g/L yeast extract, 20 g/L peptone, 20 g/L glucose) or in modified YPD medium (YPDm, 10 g/L yeast extract, 20 g/L tryptone, 20 g/L glucose). The low nitrogen content YPD contained 5 g/L yeast extract, 10 g/L tryptone, 30 g/L glucose. Synthetic dextrose medium lacking uracil and leucine (SD/(-Leu/-Ura)), 6.7 g/L yeast nitrogen base without amino acids, 0.62 g/L dropout supplement lacking leucine, tryptophan and uracil (DO/(-Leu/-Trp/-Ura)), 20 g/L glucose, 5 g/L (NH_4_)_2_SO_4_, supplemented with 100 mg/L tryptophan) was used for the selection of the integrated strains containing the assembled biochemical pathway. Synthetic dextrose medium lacking uracil (SD/-Ura) was obtained by adding 20 mg/L leucine and 100 mg/L tryptophan to the SD/(-Leu/-Trp/-Ura) medium. For the optimization of carbon sources, the yeast extract peptone (YP) medium (10 g/L yeast extract, 20 g/L peptone) was supplemented with 20 g/L glycerol or soluble starch or sucrose or oleic acid. During the construction the yeast strains were incubated at 30 °C under shacking (250 rpm). For plate cultivation, 2% agar was added to the culture medium. For β-ionone fermentation, the yeast strains were cultured in 50-mL shake-flasks containing 20 mL YPD medium and 10% (v/v) of dodecane at 20 °C, under shaking at 250 rpm.

### Construction of plasmids and strains

All the plasmids are listed in Table 2. The primers used for the construction of these plasmids are listed in Additional file 2: Table S2. The original CRISPR–Cas9 mediated genome editing plasmid pCAS1yl was a kind gift from Prof. Sheng Yang [28]. The 20-nt sequence at the 5′-end of the gRNA was modified for the gene unit integration by using the oligo DNA listed in Additional file 2: Table S2. The sequences of *carB* and *carRP* genes from *Mucor circinelloides* [36] and the sequence of *PhCCD1* gene from *Petunia hybrida* [51] were codon-usage optimized and synthesized by Sangon Biotech Co. Ltd. (Shanghai, China). All the homologous arms, promoters, terminators and endogenous genes were amplified from *Y. lipolytica* genomic DNA. Three endogenous promoters (*P*_*TEF1*_, *P*_*EXP1*_, *P*_*GPD2*_) were separately used in this work. All the seven integration units (Fig. 1b) were flanked by two 600–1100 bp homologous arms. The selection maker cassette hisG-Ura3-hisG (HUH), which was a kind gift from Prof. Sheng Yang [36], was used for gene unit integration. The DNA fragments were assembled by Gibson assembly [27]. *Not* I digested sites were used for the linearization of the units. The transformation of the plasmids and the linear DNA fragments into *Y. lipolytica* was performed using the Frozen-EZ Yeast Transformation II kit (Zymo Research Corporation). The transformation mix was spread on the selection plate and *Y. lipolytica* was cultured at 30 °C for 4 days. Detailed information for the plasmid construction was is shown in Additional file 3.

**Table 2:** The plasmids used in this work

Name	Relative characteristics	Source
pUC19		TaKaRa
pTAs-HUH	*HisG*-*Ura3*-*HisG* maker recycled cassette	[36]
pCAS1yl	*TRP1* Guide RNA module and Cas9 expression cassette in pMCSCen1	[36]
pCAS1yl-ku70	*Ku70* Guide RNA module and Cas9 expression cassette in pMCSCen1	This work
pCAS1yl-ku80	*Ku80* Guide RNA module and Cas9 expression cassette in pMCSCen1	This work
pCAS1yl-rDNA	*rDNA* Guide RNA module and Cas9 expression cassette in pMCSCen1	This work
pCAS1yl-D17	*D17* Guide RNA module and Cas9 expression cassette in pMCSCen1	This work
pCAS1yl-lip1	*Lip1* Guide RNA module and Cas9 expression cassette in pMCSCen1	This work
pCAS1yl-pox3	*Pox3* Guide RNA module and Cas9 expression cassette in pMCSCen1	This work
pCAS1yl-pox4	*Pox4* Guide RNA module and Cas9 expression cassette in pMCSCen1	This work
pCAS1yl-pox5	*Pox5* Guide RNA module and Cas9 expression cassette in pMCSCen1	This work
pCAS1yl-LEU2	*LEU2* Guide RNA module and Cas9 expression cassette in pMCSCen1	This work
pCAS1yl-XPR2	*XPR2* Guide RNA module and Cas9 expression cassette in pMCSCen1	This work
pUC19-TBX	*P*_*TEF1*_-*CarB*-*xpr2t* cassette in pUC19	This work
pUC19-ERPL	*P*_*EXP1*_-*CarRP*-*lip2t* cassette in pUC19	This work
pUC19-GCM	*P*_*GPD2*_-*CCD1*-*mig1t* cassette in pUC19	This work
pUC19-TGX	*P*_*TEF1*_-*GGS1*-*xpr2t* cassette in pUC19	This work
pUC19-GTM	*P*_*EXP1*_-*tHMG1*-*lip2t* cassette in pUC19	This work
pUC19-rDNA-BPC	*P*_*TEF1*_-*CarB*-*xpr2t* and *P*_*EXP1*_-*CarRP*-*lip2t* and *P*_*GPD2*_-*CCD1*-*mig1t* cassettes in pUC19-rDNA-HUH	This work
pUC19-ku70-BPC	*P*_*TEF1*_-*CarB*-*xpr2t* and *P*_*EXP1*_-*CarRP*-*lip2t* and *P*_*GPD2*_-*CCD1*-*mig1t* cassettes in pUC19-ku70-HUH	This work
pUC19-ku80-GGS1-HUH	*P*_*TEF1*_-*GGS1*-*xpr2t* cassette in pUC19-ku80-HUH	This work
pUC19-D17-tHMG1-HUH	*P*_*EXP1*_-*tHMG1*-*lip2t* cassette in pUC19-D17-HUH	This work
pUC19-lip1-ERG10ERG13-HUH	*P*_*TEF1*_-*ERG10*-*erg10t* and *P*_*GPD2*_-*ERG13*-*erg13t* cassettes in pUC19-lip1-HUH	This work
pUC19-POX3-IDIERG20-HUH	*P*_*EXP1*_-*IDI*-*idit* and *P*_*TEF1*_-*ERG20*-*erg20t* cassettes in pUC19-pox3-HUH	This work
pUC19-POX5-ERG8ERG12ERG19-HUH	*P*_*EXP1*_-*ERG8*-*erg8t*, *P*_*TEF1*_-*ERG12*-*erg12t* and *P*_*GPD2*_-*ERG19*-*erg19t* cassettes in pUC19-pox5-HUH	This work
pUC19-rDNA-BB-PK–PTA-HUH	*P*_*GPD2*_-*B. b pk*-*mig1t*-*P*_*TEF1*_-*B. s pta*-*xpr2t* cassettes in pUC19-rDNA-HUH	This work
pUC19-rDNA-BC-PK–PTA-HUH	P_GPD2_-B. b pk-mig1t-P_TEF1_-C. k pta-xpr2t cassettes in pUC19-rDNA-HUH	This work
pUC19-rDNA-LB-PK–PTA-HUH	*P*_*GPD2*_-*L. m pk*-*mig1t*-*P*_*TEF1*_-*B. s pta*-*xpr2t* cassettes in pUC19-rDNA-HUH	This work
pUC19-rDNA-LC-PK–PTA-HUH	*P*_*GPD2*_-*L. m pk*-*mig1t*-*P*_*TEF1*_-*C. k pta*-*xpr2t* cassettes in pUC19-rDNA-HUH	This work
pUC19-POX4-BB-PK–PTA-HUH	*P*_*GPD2*_-*B. b pk*-*mig1t*-*P*_*TEF1*_-*B. s pta*-*xpr2t* cassettes in pUC19-POX4-HUH	This work
pUC19-XPR2-BB-PK–PTA-HUH	*P*_*GPD2*_-*B. b pk*-*mig1t*-*P*_*TEF1*_-*B. s pta*-*xpr2t* cassettes in pUC19-XPR2-HUH	This work

Other plasmids can be found in Additional file 3

To generate *Y. lipolytica* Po1f/(△*ku70*△*ku80*), named YLBI0003 in this study, two CRISPR/Cas9 plasmids targeting *ku70* and *ku80* genes, respectively, were co-transformed with corresponding homologous arm fragments into *Y. lipolytica* Po1f. Eight gene loci (*rDNA*, *pox3*, *pox5*, *lip1*, *D17*, *xpr2*, *ku70* and *ku80*) of the genome were selected for the integration of these units [52–54]. The Module 3 (the β-ionone synthesis module) was first integrated at the *rDNA* locus of strain YLBI0003 to generate strain YLBI0004. Five units of the Module 2 (the MVA module) including 9 endogenous genes (*ERG10 *+ *ERG13*, *tHMG1*, *ERG8 *+ *ERG12 *+ *ERG19, IDI *+ *ERG20* and *GGS1)* were then integrated into five loci (*lip1*, *D17*, *pox5*, *pox3* and *ku80*) of YBLI0004 with different unit combinations (Fig. 1a). The Module 1 (the acetyl-CoA supply module) was subsequently integrated into the *xpr2* locus of strain YLBI3017 to generate strain YLBI3117. Another copy of Module 3 was also integrated at the *ku70* locus of strain YLBI3117 to generate strain YLBI3118. All the strains constructed in this study are listed in Table 3. KOD FX DNA polymerase (Toyobo Co; Osaka, Japan) was used for colony PCR to confirm the strain genotypes using the primers listed in Additional file 2: Table S3. The PCR products were sequenced at Sangon Biotech Co. Ltd. (Shanghai, China). The pCAS1yl-gRNA plasmid was simply cured by picking out the single colonies followed by culturing in YPD medium for about 16 h until the OD600 was approximately 3 and then plating onto YPD agar with a 1000-fold dilution. The Ura3 selection maker was cured by incubating the strain on YPD plate containing 1 mg/mL 5-fluoroorotic acid for 2–3 days. The larger colonies were then streaked onto SD and SD/-Ura plates and were cultured at 30 °C for 2 more days.

**Table 3:** *Y. lipolytica* strains constructed in this work

strains	Characteristics	Source
CLIB 724/po1f	*MatA, leu2*-*270, ura3*-*302, xpr2*-*322, axp1*-*2 Leu*-*, Ura*-*, DAEP, DAXP, Suc*+	[39]
YLBI0001	CLIB 724 *△ku70*::*hisG*	This work
YLBI0002	CLIB 724 *△ku80*::*hisG*	This work
YLBI0003	YLBI0001 *△ku80*::*hisG*	This work
YLBI0004	YLBI0003 *△rDNA*::*P*_*TEF1*_-*CarB*-*xpr2t*, *P*_*EXP1*_-*CarRP*-*lip2t*, *P*_*GPD2*_-*CCD1*-*mig1t*	This work
YLBI1001	YLBI0004 *△KU80*::*P*_*TEF1*_-*GGS1*-*xpr2t*	This work
YLBI2001	YLBI0004 *△D17*::*P*_*EXP1*_-*tHMG1*-*lip2t*	This work
YLBI3001	YLBI1001 *△D17*::*P*_*EXP1*_-*tHMG1*-*lip2t*	This work
YLBI3011	YLBI3001 *△lip1*:*:P*_*TEF1*_-*ERG10*-*erg10t*, *P*_*GPD2*_-*ERG13*-*erg13t*	This work
YLBI3012	YLBI3001 *△Pox3*::*P*_*EXP1*_-*IDI*-*idit*, *P*_*TEF1*_-*ERG20*-*erg20t*	This work
YLBI3013	YLBI3001 *△Pox5*::*P*_*EXP1*_-*ERG8*-*erg8t*, *P*_*TEF1*_-*ERG12*-*erg12t*, *P*_*GPD2*_-*ERG19*-*erg19t*	This work
YLBI3014	YLBI3011 *△Pox3*::*P*_*EXP1*_-*IDI*-*idit*, *P*_*TEF1*_-*ERG20*-*erg20t*	This work
YLBI3015	YLBI3011 *△Pox5*::*P*_*EXP1*_-*ERG8*-*erg8t*, *P*_*TEF1*_-*ERG12*-*erg12t*, *P*_*GPD2*_-*ERG19*-*erg19t*	This work
YLBI3016	YLBI3012 *△Pox5*::*P*_*EXP1*_-*ERG8*-*erg8t*, *P*_*TEF1*_-*ERG12*-*erg12t*, *P*_*GPD2*_-*ERG19*-*erg19t*	This work
YLBI3017	YLBI3014 *△Pox5*::*P*_*EXP1*_-*ERG8*-*erg8t*, *P*_*TEF1*_-*ERG12*-*erg12t*, *P*_*GPD2*_-*ERG19*-*erg19t*	This work
YLBI0101	YLBI0003 *△rDNA*::*P*_*GPD2*_-*B. b pk*-*mig1t*-*P*_*TEF1*_-*B. s pta*-*xpr2t*	This work
YLBI0102	YLBI0003 *△rDNA*::*P*_*GPD2*_-*B. b pk*-*mig1t*-*P*_*TEF1*_-*C. k pta*-*xpr2t*	This work
YLBI0103	YLBI0003 *△rDNA*::*P*_*GPD2*_-*L. m pk*-*mig1t*-*P*_*TEF1*_-*B. s pta*-*xpr2t*	This work
YLBI0104	YLBI0003 *△rDNA*::*P*_*GPD2*_-*L. m pk*-*mig1t*-*P*_*TEF1*_-*C. k pta*-*xpr2t*	This work
YLBI3110	YLBI3001 *△rDNA*::*P*_*GPD2*_-*B. b pk*-*mig1t*-*P*_*TEF1*_-*B. s pta*-*xpr2t*	This work
YLBI3111	YLBI3001 *△POX4*::*P*_*GPD2*_-*B. b pk*-*mig1t*-*P*_*TEF1*_-*B. s pta*-*xpr2t*	This work
YLBI3112	YLBI3001 *△XPR2*::*P*_*GPD2*_-*B. b pk*-*mig1t*-*P*_*TEF1*_-*B. s pta*-*xpr2t*	This work
YLBI3113	YLBI3111 *△POX4*::*P*_*GPD2*_-*B. b pk*-*mig1t*-*P*_*TEF1*_-*B. s pta*-*xpr2t*	This work
YLBI3114	YLBI3111 *△XPR2*::*P*_*GPD2*_-*B. b pk*-*mig1t*-*P*_*TEF1*_-*B. s pta*-*xpr2t*	This work
YLBI3115	YLBI3112 *△XPR2*::*P*_*GPD2*_-*B. b pk*-*mig1t*-*P*_*TEF1*_-*B. s pta*-*xpr2t*	This work
YLBI3116	YLBI3113 *△XPR2*::*P*_*GPD2*_-*B. b pk*-*mig1t*-*P*_*TEF1*_-*B. s pta*-*xpr2t*	This work
YLBI3117	YLBI3017 *△rDNA*::*P*_*GPD2*_-*B. b pk*-*mig1t*-*P*_*TEF1*_-*B. s pta*-*xpr2t*	This work
YLBI3118	YLBI3117 *△ku70*::*P*_*TEF1*_-*CarB*-*xpr2t, P*_*EXP1*_-*CarRP*-*lip2t, P*_*GPD2*_-*CCD1*-*mig1t*	This work

### Integration verification

All the primers for colony PCR verification are listed in Additional file 2: Table S3. Briefly, the *ku70* and *ku80* knockout strains were confirmed by colony PCR using the ku70-CP-F/R and ku80-CP-F/R primer pairs. The strain integrated with the Module 3 for the β-ionone synthesis was first picked out based on the yellow phenotype and the genotype was verified by PCR using CarB-CP-F/R, CarRP-CP-F/R and CCD1-CP-F/R primer pairs. All the other integrated strains were confirmed with the relevant primers. The PCR products were sequenced at Sangon Biotech Co. Ltd. (Shanghai, China). Real-time qPCR was performed to determine the copy number of the inserted units using the TB Green™ Premix Ex Taq™ kit (TaKaRa, Dalian, China). The PCR reaction was monitored on an ABI 7500 real Time PCR System. The *act1* homologous genes were chosen as reference.

### Fed-batch fermentations in 3-L bioreactor

The initial fermentation was completed using 1.2 L YPDm medium containing 20 g/L glucose, 20 g/L tryptone, 10 g/L yeast extract in 3-L bioreactor (BioFlo/CelliGen 115, New Brunswick, Canada). The seed culture was incubated in a shake-flask at 30 °C, 250 rpm for 16 h and inoculated into the bioreactor at an initial OD ~ 0.2. Dodecane (150 mL, 10% v/v) was used as the initial organic overlay layer. 250 mL 10× YPDm was fed at 0.4 mL/min after 12 h fermentation, followed by 600 g/L glucose at 0.1 mL/min till the end of the fermentation. The temperature was maintained at 20 °C throughout the fermentation. The pH was maintained at 5.5 by the automatic addition of 3 M NaOH or 3 M HCl. The dissolved oxygen (DO) was set at 0–5%, 15%, 25%, 35% under automatic control of agitation speed (100–1000 rpm), and the aeration was set at 2 L/min. Pure oxygen gas was essential to achieve high DOs.

### Biomass, sugar and organic acid quantification

A 1 mL aliquot of cultured cells was harvested and dried at 60 °C for 48 h to measure the DCW. Acetate, glucose and other carbon sources were filtered by through 0.2 μm pore size membrane and then quantified by high-performance liquid chromatography (HPLC) using an Agilent1260 Infinity series system (Agilent Technologies, USA) equipped with an Aminex HPX 87H column (300 mm × 7.8 mm, Bio-Rad Laboratories, USA) and a refractive index detector set at 210 nm. The analytes were eluted with 5 mM H_2_SO_4_ at 60 °C at 0.6 mL/min flow rate.

### β-ionone quantification

The organic phase was carefully pipetted from the culture sample and centrifuged for 5 min at 12,000 rpm. β-ionone in the aqueous medium and cell pellets were extracted using dodecane as described in literature [30]. The supernatant organic phase filtered by through 0.2 μm pore size membrane and analyzed by injecting a 1 µL sample into a gas chromatography system HP 7890A (Agilent Technologies, USA) coupled to a flame ionization detector using a DB-FFAP capillary column (60 m × 0.25 mm id, 0.25 µm film thickness) (J&W Scientific, Agilent Technologies, USA). The oven program was set as follows: start at 80 °C for 1 min, then the temperature was raised up 10 °C/min to 120 °C and kept constant for 1 min then raised 10 °C/min to 240 °C. The standard curve was constructed within the range of 1 mg/L-100 mg/L β-ionone. Isolongifolene (Sigma-Aldrich, USA) was used as internal standard.

### β-carotene quantification

For β-carotene extraction, 0.1 mL cultured cells were harvested by centrifugation for 5 min at 12,000 rpm. A 0.7 mL aliquot of dimethyl sulfoxide was used to resuspend the cells followed by an incubation at 55 °C for 10 min. After addition of 0.7 mL acetone, the sample was incubated at 45 °C for 15 min. The sample was centrifuged at 13,000*g* for 5 min. The supernatant was filtered by through 0.2 μm pore size membrane and then analyzed by HPLC using an Agilent 1260 Infinity serious system (Agilent Technologies, USA) with an UV detector (wavelength 450 nm) and an XDB-C18 column (5 μm, 4.6 × 150 mm, Agilent Technologies, USA). The mobile phase consisted of methanol, acetonitrile and dichloromethane (42:42:16) with a flow rate of 1.0 mL/min at 30 °C.

## Supplementary information

**Additional file 1: Figures S1–S10.**

**Additional file 2: Table S1–S3.**

**Additional file 3.** Methods for plasmids construction.

## Data Availability

All data generated or analyzed during this study are included in this published article and its additional information files.

## Abbreviations

PK: Phosphoketolase
PTA: Phosphotransacetylase
ERG10: Acetoacetyl-CoA thiolase
ERG13: Hydroxymethylglutaryl-CoA synthase
tHMGR: Truncated hydroxymethylglutaryl-CoA reductase
ERG8: Phosphomevalonate kinase
ERG12: Mevalonate kinase
ERG19: Mevalonate diphosphate decarboxylase
IDI: Isopentenyl diphosphate isomerase
ERG20: Geranyl/farnesyl diphosphate synthase
GGS1: GGPP synthase
carB: Phytoene dehydrogenase
carRP: Phytoene synthase/lycopene cyclase
CCD1: Carotenoid cleavage dioxygenase
HMG-CoA: Hydroxymethylglutaryl-CoA
IPP: Isopentenyl diphosphate
DMAPP: Dimethylallyl diphosphate
FPP: Farnesyl diphosphate
GGPP: Geranylgeranyl diphosphate
*Y. lipolytica*: *Yarrowia lipolytica*
HR: Heterologous recombination
HUH: HisG-Ura3-HisG
YPD: Yeast extract-peptone-dextrose
DCW: Dry cell weight
DO: Dissolved oxygen
C/N: Ratio of carbon resource to nitrogen resource
HPLC: High-performance liquid chromatography
