# Supplementary material for: A modular pathway engineering strategy for the high-level production of β-ionone in Yarrowia lipolytica
Source: Microb Cell Fact. 2020 Feb 27;19:49. doi: 10.1186/s12934-020-01309-0 (PMC7045511; doi:10.1186/s12934-020-01309-0)
Supplement: Supplementary file 1 — Additional file 1: Figures S1–S10. [file 12934_2020_1309_MOESM1_ESM.docx]

**Additional file 1**


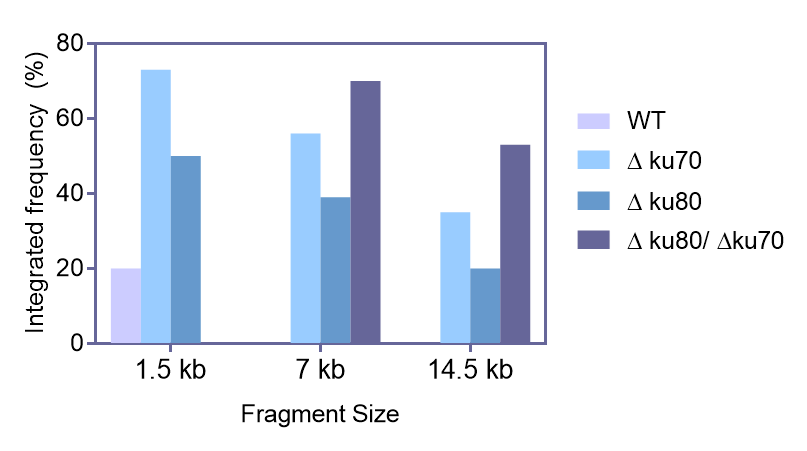


**Figure S1.** CRISPR-Cas9 mediated the integration of different size fragments at *rDNA* locus in △*ku70* and/or △*ku80* disrupted strains. The wild type *Y. lipolytica* po1f strain was used as native control and the integration frequency of 7 kb or 14.5 kb in wild type strain was undetectable. The 1.5 kb, 7 kb and 14.5 kb fragments represented: the homologous arm from the *ku70* or *ku80* loci, GFP integrated cassette and β-ionone biosynthesis cassette, respectively.


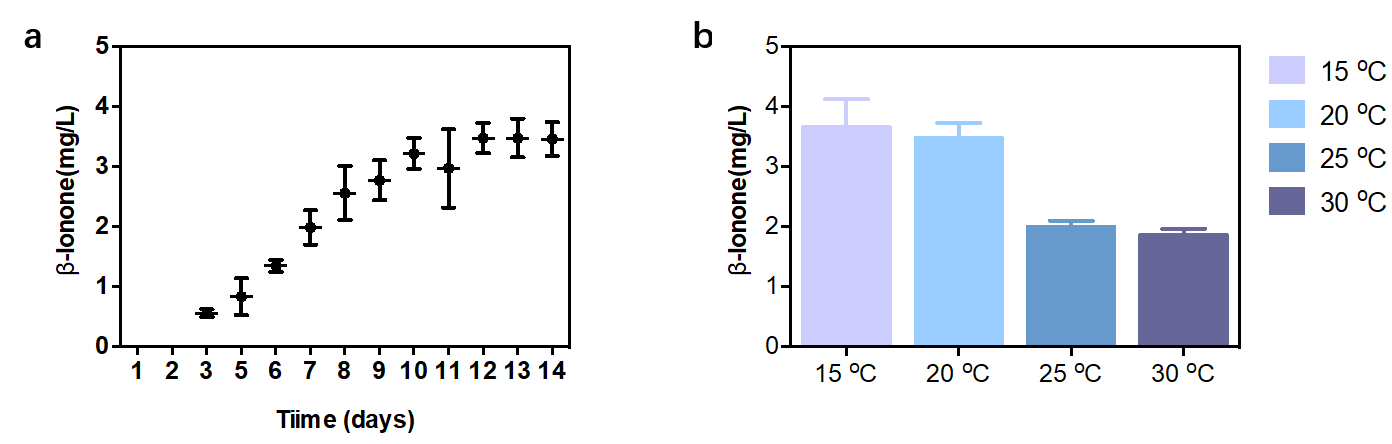


**Figure S2.** β-ionone production at different temperatures. **a)** β-ionone production in flask fermentation at 15 ^o^C, 20 ^o^C, 25 ^o^C, 30 ^o^C. **b**) β-ionone production in flask fermentation versus fermentation time at 20 ^o^C; Data represent the mean ± standard deviation (n=3).


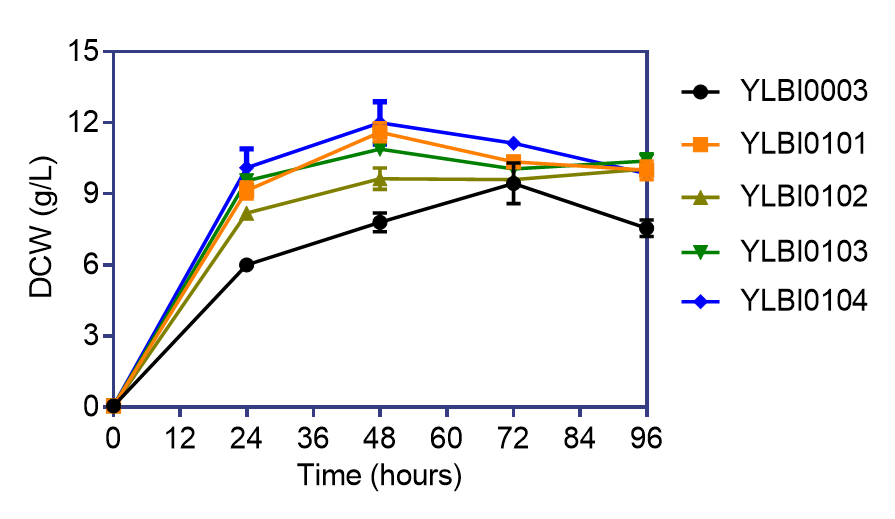


**Figure S3.** The introduction of the PK-PTA pathway correlated with an increased growth and biomass formation. *Y. lipolytica* po1f was used as the native control. *lmPK* from *Leuconostoc mesenteroides*, *bbPK* from *Bifidobacterium bifidum,* *ckPTA* from *Clostridium kluyveri*; *bsPTA* from [*Bacillus subtilis*](http://www.so.com/link?url=http://dict.youdao.com/search?q=bacillus%20subtilis&keyfrom=hao360&q=bacillus+subtilis&ts=1513597254&t=77694570933a8c5a595ca1ed520f3bb). Data represent the mean ± standard deviation (n=3).


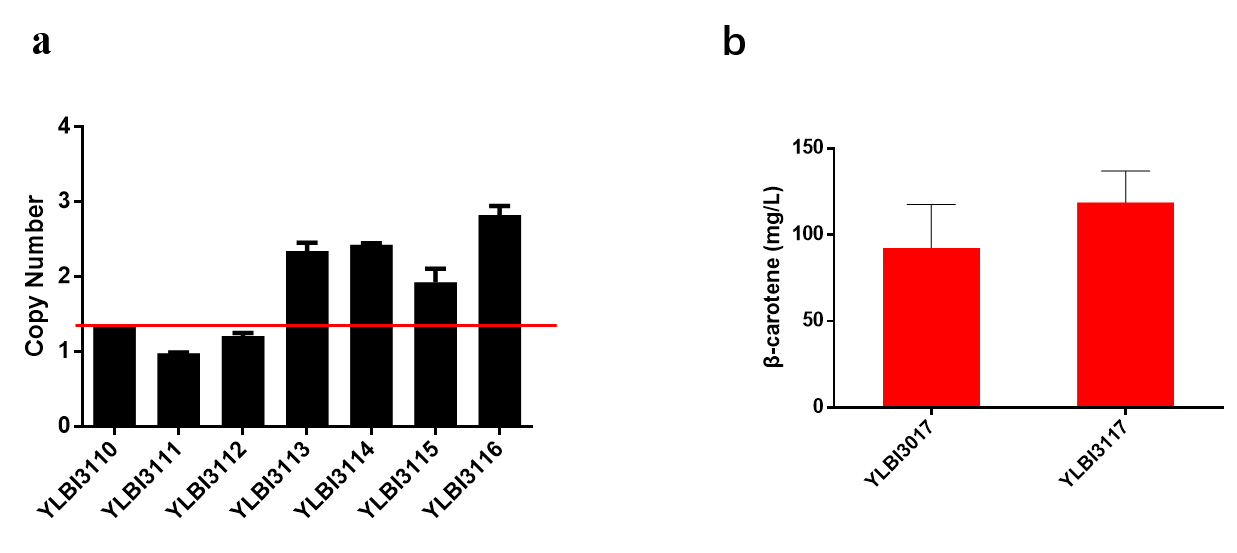


**Figure S4. a)** Copy number analysis of *bbPK-bsPTA* in different engineered *Y. lipolytica* transformants by qPCR. The *act1* homologous gene were chosen as reference. **b)** β-carotene production in engineered strains integrated with different copy numbers of *bbPK-bsPTA*. Data represent the mean ± standard deviation (n=3).


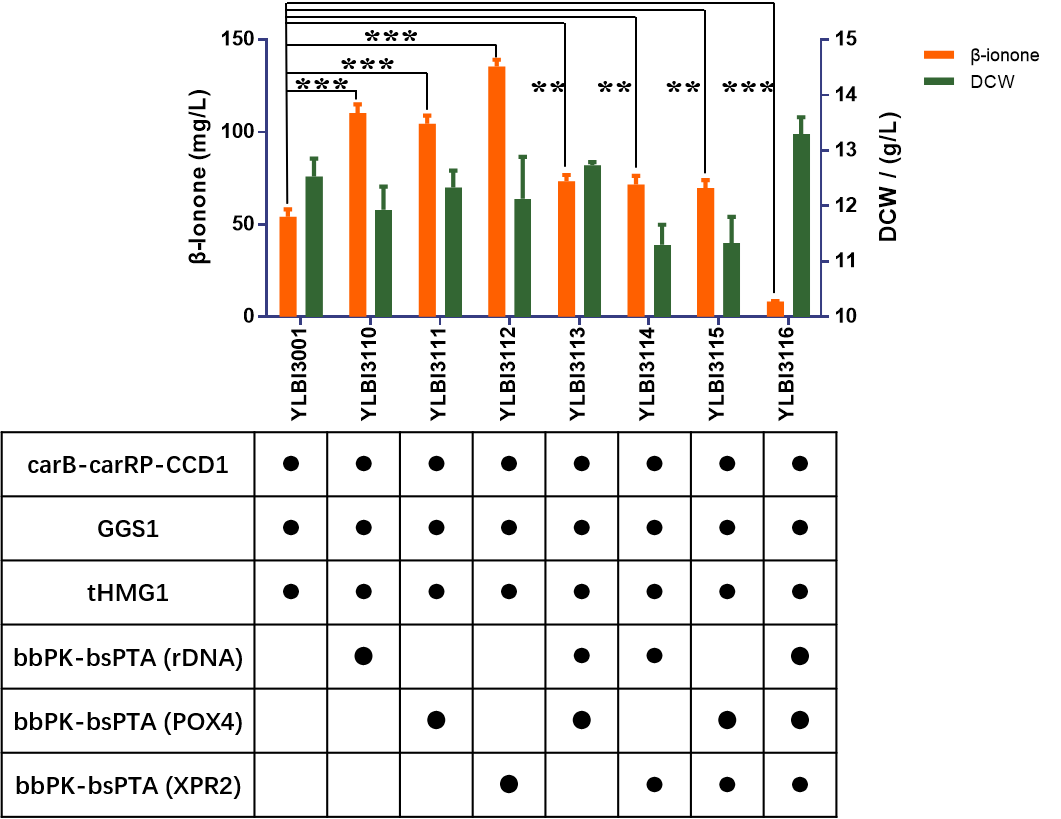


**Figure S5.** β-ionone production in engineered strains that integrated with different number of *bbPK-bsPTA*. Significance was marked by t-test, *p-value ≤0.05; **p-value ≤0.01; ***p-value ≤0.001.


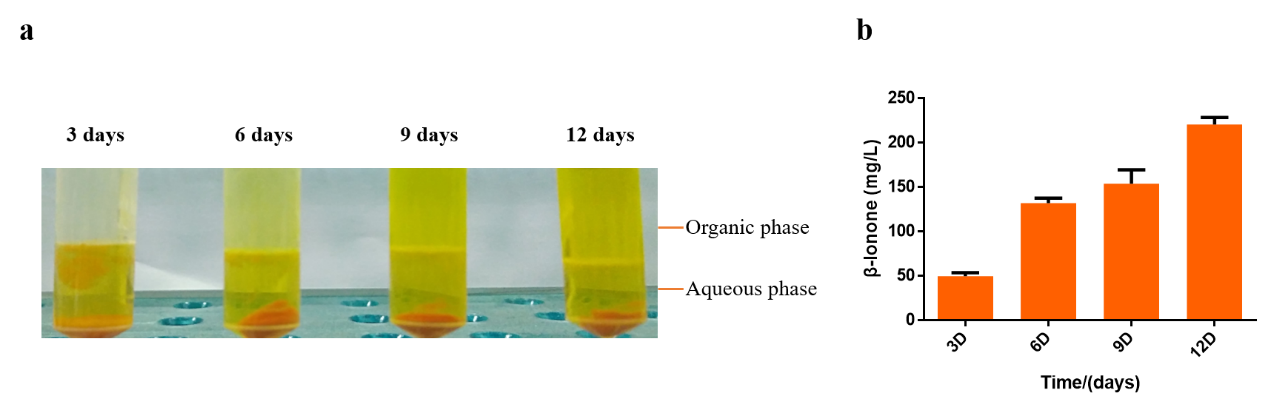


**Figure S6.** Flask fermentation results. **a)** the color change of the organic phase versus fermentation time (fermentation performed at 20 ^o^C). **b)** β-ionone production versus fermentation time obtained with strain YLBI3118 in flask fermentation. Data represent the mean ± standard deviation (n=3).


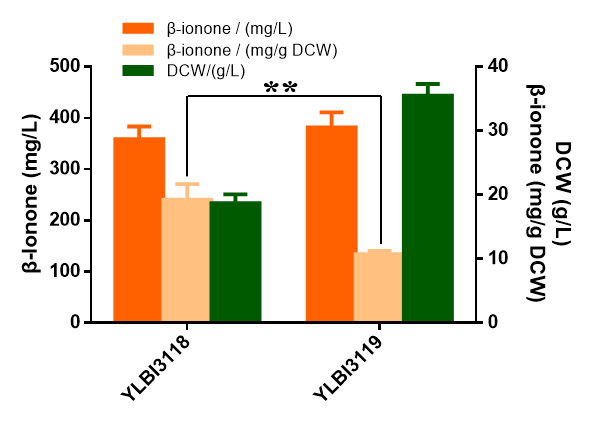


**Figure S7.** Production of β-ionone from flask fermentation by engineered strains YLBI3118 and YLBI3119*.* Data represent the mean ± standard deviation (n=3). Significance was marked by t-test, **p-value ≤0.01.


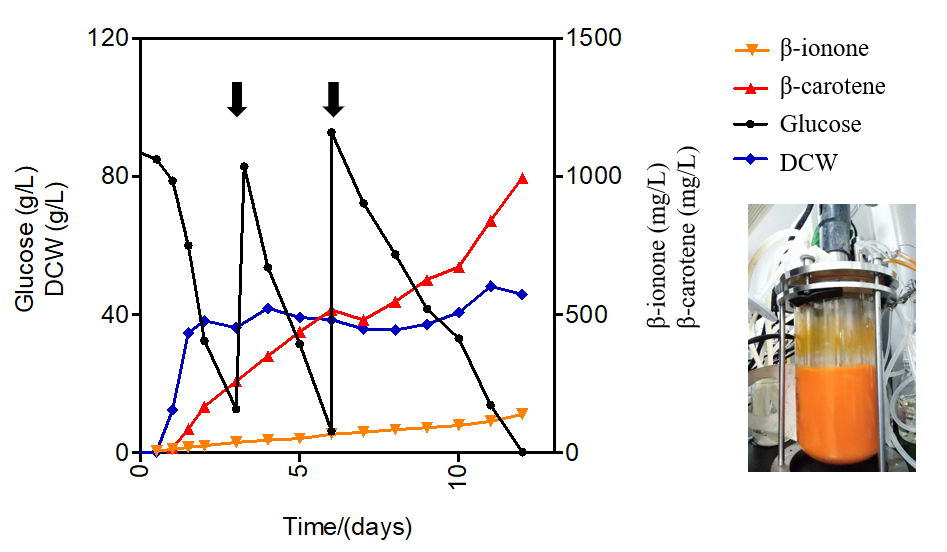


**Figure S8.** Production of β-ionone by engineered strain YLBI3118 in bioreactor fermentation. The black arrow indicates the feeding of glucose. The picture of bioreactor represents the culture color at the end point of the fermentation.


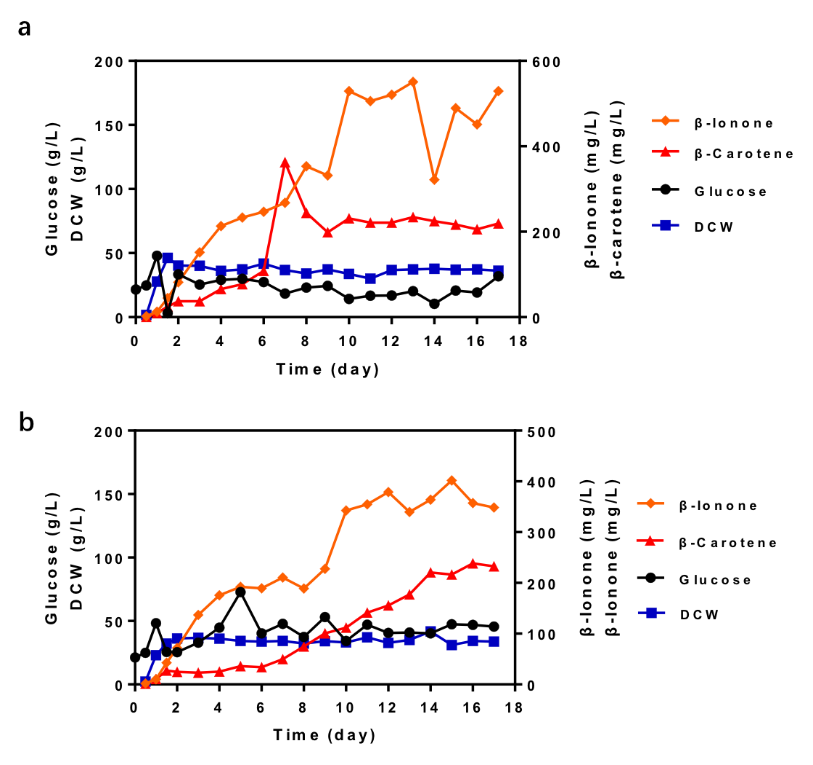


**Figure S9.** Production of β-ionone by engineered strain YLBI3118 in bioreactor fermentation obtained with **a**) 25% DO and **b**) 35% DO.


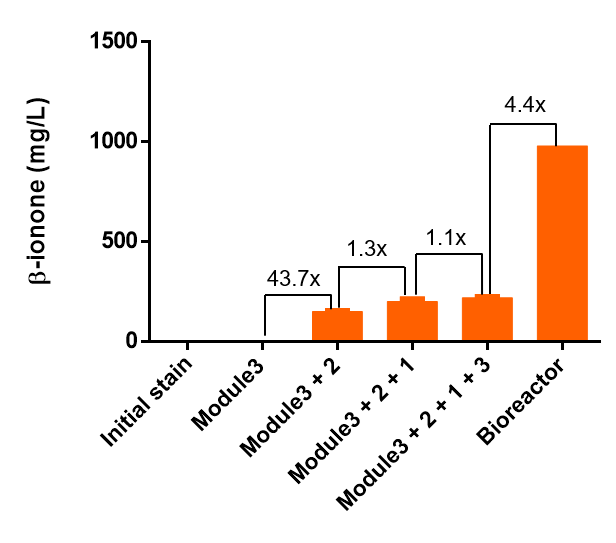


**Figure S10**: The β-ionone titers change versus different modules or bioreactor fermentation.
