# Supplementary material for: A modular pathway engineering strategy for the high-level production of β-ionone in Yarrowia lipolytica
Source: Microb Cell Fact. 2020 Feb 27;19:49. doi: 10.1186/s12934-020-01309-0 (PMC7045511; doi:10.1186/s12934-020-01309-0)
Supplement: Supplementary file 2 — Additional file 2: Table S1–S3. [file 12934_2020_1309_MOESM2_ESM.docx]

**Additional file 2**

**Table S1.** Summary of β-ionone production from microorganisms.

| Host | Titer/(g/L) | Productivity /(mg/L/h） | Reference |
| --- | --- | --- | --- |
| *E. coli* | 0.5 | 10 | [1] |
| *S. cerevisiae* | 0.18 | 2.5 | [2] |
| *Y. lipolytica* | 0.38 | 2.7 | [3] |
| *Y. lipolytica* | 0.98 | 2.4 | This work |

**Table S2**. Primers used in this study for the construction of plasmids and the confirmation of transformants.

| Name | Sequence (5` to 3`) |
| --- | --- |
| pCAS1yl-F | TGTGACCGTCTCCGGGAGC |
| pCAS1yl-R | GCTCCCGGAGACGGTCACA |
| ku70-gRNA-F | AACTCTTCATAAGGCCTTGGGTTTTAGAGCTAGAAATAGCA |
| ku70-gRNA-R | CCAAGGCCTTATGAAGAGTTGACGAGCTTACTCGTTTCGT |
| ku80-gRNA-F | TCCTAGCCAGAACAACCTTCGTTTTAGAGCTAGAAATAGCA |
| ku80-gRNA-R | GAAGGTTGTTCTGGCTAGGAGACGAGCTTACTCGTTTCGT |
| rDNA-gRNA-F | GGAGTAACTATGCTCTCTTAGTTTTAGAGCTAGAAATAGCA |
| rDNA-gRNA-R | TAAGAGAGCATAGTTACTCCGACGAGCTTACTCGTTTCGT |
| gRNA-D17-F | TCCGTAATATAGGTGACGACGTTTTAGAGCTAGAAATAGCA |
| gRNA-D17-R | GTCGTCACCTATATTACGGAGACGAGCTTACTCGTTTCGT |
| gRNA-pox5-F | CCTCTGACTTCACCCTATCCGTTTTAGAGCTAGAAATAGCA |
| gRNA-pox5-R | GGATAGGGTGAAGTCAGAGGGACGAGCTTACTCGTTTCGT |
| gRNA-pox4-F | GAGTTGACGAGAACTGTCGTGTTTTAGAGCTAGAAATAGCA |
| gRNA-pox4-R | ACGACAGTTCTCGTCAACTCGACGAGCTTACTCGTTTCGT |
| gRNA-pox3-F | CCCTTGTACCGGTAGCTAATGTTTTAGAGCTAGAAATAGCA |
| gRNA-pox3-R | ATTAGCTACCGGTACAAGGGGACGAGCTTACTCGTTTCGT |
| gRNA-lip1-F | GCTCGGCAACCAGGAATGGAGTTTTAGAGCTAGAAATAGCA |
| gRNA-lip1-R | TCCATTCCTGGTTGCCGAGCGACGAGCTTACTCGTTTCGT |
| gRNA-XPR2-F | GCTGGACTCTCTGGTCGACGGTTTTAGAGCTAGAAATAGCA |
| gRNA-XPR2-R | CGTCGACCAGAGAGTCCAGCGACGAGCTTACTCGTTTCGT |
| pUC19-tong-F | GAATTCACTGGCCGTCGTTTTA |
| pUC19-tong-R | GGCGTAATCATGGTCATAGCTG |
| rDNA-up-NotI-F | GCTATGACCATGATTACGCCACGCGTGCGGCCGCATCGATCCTAAGGGGTGGCAT |
| rDNA-down-NotI-R | AAACGACGGCCAGTGAATTCACGCGTGCGGCCGCCTTCGGTATGATAGGAAGAG |
| Ku70-up-NotI-F | GCTATGACCATGATTACGCCACGCGTGCGGCCGCTGTTTCAAATCAGCCTGTCGTTT |
| Ku70-down-NotI -R | AAACGACGGCCAGTGAATTCACGCGTGCGGCCGCGTGAAAGGAACATAGTCATTT |
| Ku80-up-NotI -F | GCTATGACCATGATTACGCCACGCGTGCGGCCGCTAATTGAACTCACTTCTTTGG |
| Ku80-down-NotI -R | AAACGACGGCCAGTGAATTCACGCGTGCGGCCGCTCGCTTCTTG GCTTCTCTAAA |
| D17-NotI-up-F | GCTATGACCATGATTACGCCACGCGTGCGGCCGCATAGCTCGCTCGAACTGCCAC |
| D17-NotI-down-R | AAACGACGGCCAGTGAATTCACGCGTGCGGCCGC CCACTATATCACCCCTCCAAC |
| Xpx2-NotI-up-F | GCTATGACCATGATTACGCCACGCGTGCGGCCGC CATTATCGAGACCGTTGTTCC |
| Xpx2-NotI-down-R | AAACGACGGCCAGTGAATTCACGCGTGCGGCCGCGGTGAGAACCTCGTCATTGAT |
| Lip1-NotI-up-F | GCTATGACCATGATTACGCCACGCGTGCGGCCGCCGTCGCTCATGGAAAAGCCC |
| Lip1-NotI-down-R | AAACGACGGCCAGTGAATTCACGCGTGCGGCCGCAGCACCTGCAACTGGTGCATG |
| Pox5-NotI-up-F | GCTATGACCATGATTACGCCACGCGTGCGGCCGCACACCGAACCTGGTCGTCTAC |
| Pox5-NotI-down-R | AAACGACGGCCAGTGAATTCACGCGTGCGGCCGCAAATGTTCATTGACGTGTCCAT |
| Pox3-NotI-up-F | GCTATGACCATGATTACGCCACGCGTGCGGCCGCAGCGCAAGTTTCAGCGCTC |
| Pox3-NotI-down-R | AAACGACGGCCAGTGAATTCACGCGTGCGGCCGCGCCAAGATCATGTGATTATGG |
| Pox4-NotI-up-F | GCTATGACCATGATTACGCCACGCGTGCGGCCGCATATCCTCGGGCTCCATGGGG |
| Pox4-NotI-down-R | AAACGACGGCCAGTGAATTCACGCGTGCGGCCGCCGAAACCCGACTAATTGACTA |
| rDNAu-R | CCCTGATTGACTGGAACAGCTTGAATTTCTTCACTTTGACATTC |
| rDNA-hisG-F | GTCAAAGTGAAGAAATTCAAGCTGTTCCAGTCAATCAGGGTA |
| Ura3-R | CCCCCTCAAGGAACTTGCTCTTAA |
| Ura3-F | GAAGAAACCGTGCTTAAGAGCAAGT |
| rDNA-hisG-R | GATGACGAGGCATTTGGCTACGGATCTTCCAGTGGTGCATGAA |
| rDNAd-F | ATGCACCACTGGAAGATCCGTAGCCAAATGCCTCGTCATCTA |
| pUC19-GGS1-F | TGCGCAGTGACCTGTCCCCACGTTGCCGGTC |
| pUC19-GGS1-R | TATAATCCATTTTGAATGATTCTTATACTCAGA |
| GGS1-P-F | ATCATTCAAAATGGATTATAACAGCGCGGATTT |
| GGS1-t-R | TGGGGACAGGTCACTGCGCATCCTCAAAGTACTT |
| Ku80-up-R | ATTGACTGGAACAGCACGGCTTTGACCTTGGGGATA |
| Ku80-hisG-F | CAAGGTCAAAGCCGT GCTGTTCCAGTCAATCAGGGTATT |
| Ku80-hisG-R | GCCAACCCGGTCTCT CGGATCTTCCAGTGGTGCATGAA |
| TEF1p-GGS1-F | CCACTGGAAGATCCGAGAGACCGGGTTGGCGGCGTAT |
| xpr2t-GGS1-R | TTTCCGCCAATCCCGTCGGACACGGGCATCTCACTT |
| Ku80-down-F | GATGCCCGTGTCCGACGGGATTGGCGGAAAGAAGGA |
| pUC19-tHMG1-F | ACGGTCATAGGCTATTTATCACTCTTTACAACTTC |
| pUC19-tHMG1-R | ACTGGGTCATTGCTGTAGATATGTCTTGTG |
| tHMG1-P-F | ATCTACAGCAATGACCCAGTCTGTGAAGGTGGTTG |
| tHMG1-t-R | GATAAATAGCCTATGACCGTATGCAAATATTCGA |
| D17-up-R | ATTGACTGGAACAGCAGTGGTACTCAAGCTCAGAAC |
| D17-hisG-F | AGCTTGAGTACCACTGCTGTTCCAGTCAATCAGGGTATT |
| D17-hisG-R | TGACCTTGGGGATATCCTCCACCTGTGTCAATCTTC |
| EXP1p-tHMG1-F | TGACACAGGTGGAGGATATCCCCAAGGTCAAAGCCG |
| lip2t-tHMG1-R | GCTAGTCTTCTATCTACGGCTTTGACCTTGGGGATA |
| D17-down-F | CAAGGTCAAAGCCGTAGATAGAAGACTAGCTTGGAC |
| pUC19-t-F | AAGCTTGGCGTAATCATGGTC |
| pUC19-TEF1p-R | TTTGAATGATTCTTATACTCAGAAG |
| pUC19-EXP2p-R | TGCTGTAGATATGTCTTGTGTG |
| pUC19-GDP2p-R | TGTTGATGTGTGTTTAATTCAAG |
| ERG10-F | CGAGTATAAGAATCATTCAAAATGGAGCCCGTCTACATTGTTT |
| ERG10-R | GACCATGATTACGCCAAGCTTTGTCAGCCCTGGTCTAACGAA |
| ERG13-F | GACCATGATTACGCCAAGCTTATGTCGCAACCCCAGAACGTT |
| ERG13-R | GACCATGATTACGCCAAGCTTGGAGTAACAGCACGTATCGCA |
| ERG12-F | CGAGTATAAGAATCATTCAAAATGGACTACATCATTTCGGC |
| ERG12-R | GACCATGATTACGCCAAGCTTTCCTCTCATTCTGGTCAAGC |
| ERG8-F | GACCATGATTACGCCAAGCTTATGACCACCTATTCGGCTCC |
| ERG8-R | GACCATGATTACGCCAAGCTTTCACTTGACTTACACCGTCCC |
| ERG19-F | GACCATGATTACGCCAAGCTTATGATCCACCAGGCCTCCAC |
| ERG19-R | GACCATGATTACGCCAAGCTTTGGAGCCCGTTGAGGGAGAT |
| ERG20-F | CGAGTATAAGAATCATTCAAAAGGACTCGGGTCAGAAGTTCT |
| ERG20-R | GACCATGATTACGCCAAGCTTTGTCCAAGGCGAAATTCGAAAG |
| IDI-F | GACCATGATTACGCCAAGCTTATGACGACGTCTTACAGCGAC |
| IDI-R | GACCATGATTACGCCAAGCTTGACTCGATACTACTCCAGTCA |
| Lip1up-R | CAACCCGGTCTCTTGCACTTTGACAGCAACTTGTA |
| ERG10-TEF1p-F | TGTCAAAGTGCAAGAGACCGGGTTGGCGGCGT |
| erg10t-ERG13-R | GGACATCCTACTGCGTGTCAGCCCTGGTCTAACGAAAA |
| ERG13-ERG10-F | AGACCAGGGCTGACACGCAGTAGGATGTCCTGCAC |
| erg13t-R | TGGAACAGCCCCGGAGTAACAGCACGTATCGCA |
| Lip1-hisG-F | GTGCTGTTACTCCGGGGCTGTTCCAGTCAATCAG |
| Lip1-hisG-R | ACTCGTCTCTTTCGGATCTTCCAGTGGTGCATGAA |
| Lip1down-F | ACTGGAAGATCCGAAAGAGACGAGTGTCCAGCTTAC |
| pox3up-R | GGCGCCAAACTCGCCTCATTTCGCGCTGTATATA |
| IDI-EXP1p-F | CGCGAAATGAGGCGAGTTTGGCGCCCGTTTTTTC |
| idit-ERG20-R | GCCAACCCGGTCTCTGACTCGATACTACTCCAGTC |
| ERG20-IDI-F | GAGTAGTATCGAGTCAGAGACCGGGTTGGCGGCGT |
| erg20t-R | TGGAACAGCCCCAGGACTCGGGTCAGAAGTTC |
| Pox3-hisG-F | TGACCCGAGTCCTGGGGCTGTTCCAGTCAATCAG |
| Pox3-hisG-R | CTGTATCATGGATTTGATCTTCCAGTGGTGCATGAA |
| Pox3down-F | CTGGAAGATCCGCATCAAATCCATGATACAGAAGACCT |
| Pox5up-R | GGCGCCAAACTCGGCAACTAAGCCTGTTGACG |
| ERG8-EXP1p-F | AGGCTTAGTTGCCGAGTTTGGCGCCCGTTTTTTC |
| erg8t-ERG12-R | GCCAACCCGGTCTCTTCACTTGACTTACACCGTCCC |
| ERG12-ERG8-F | GTGTAAGTCAAGTGAAGAGACCGGGTTGGCGGCGT |
| erg12t-ERG19-R | GGACATCCTACTGCGTCCTCTCATTCTGGTCAAGC |
| ERG19-ERG12-F | ACCAGAATGAGAGGACGCAGTAGGATGTCCTGCAC |
| erg19t-R | TGGAACAGCCCCTGGAGCCCGTTGAGGGAGAT |
| Pox5-hisG-F | TCAACGGGCTCCAGGGGCTGTTCCAGTCAATCAG |
| Pox5-hisG-R | TGGTGAACTATGCGGATCTTCCAGTGGTGCATGAA |
| Pox5down-F | ACTGGAAGATCCGCATAGTTCACCATCTTTCGGG |
| hisG-TEF1p-R | GCCAACCCGGTCTCTCGGATCTTCCAGTGGTGCATGAA |
| TEF1p-hisG-F | CCACTGGAAGATCCGAGAGACCGGGTTGGCGGCGTA |
| TEF1p-B-R | CTTCTTGGACATTTTGAATGATTCTTATACTCAGAA |
| CarB-F | AGAATCATTCAAAATGTCCAAGAAGCACATCGT |
| CarB-R | ACGTGGGGACAGGTCAAATCACGTTAGAGTTATG |
| xpr2t-B-F | AACGTGATTTGACCTGTCCCCACGTTGCCGG |
| xpr2t-EXP1p-R | AAAAAACGGGCGCCAAACTCTCGGACACGGGCATCTCACT |
| EXP1p-xpr2t-F | AGTGAGATGCCCGTGTCCGAGAGTTTGGCGCCCGTTTTTT |
| EXP1p-P-R | AGGTCAGCAGCATTGCTGTAGATATGTCTTGTG |
| CarRP-F | ATATCTACAGCAATGCTGCTGACCTACATGGA |
| CarRP-R | AGTGATAAATAGCTTAGATGGTGTTCAGGTTTC |
| lip2t-P-F | AACACCATCTAAGCTATTTATCACTCTTTACAAC |
| lip2t-GPD2p-R | GTGCAGGACATCCTACTGCGCCTCCACCTGTGTCAATCTT |
| GPD2p-lip2t-F | AAGATTGACACAGGTGGAGGCGCAGTAGGATGTCCTGC |
| GPD2p-C-R | CCTTTCTACCCATTGTTGATGTGTGTTTAATTCA |
| CCD1-F | CACACATCAACAATGGGTAGAAAGGAGTCTGAC |
| CCD1-R | GACCGGCCAGTGTTACAGCTTAGCCTGCTCCT |
| mig1t-C-F | GGCTAAGCTGTAACACTGGCCGGTCGATAATTTA |
| mig1t-rDNA-R | CATTCATGCGCGTCACTAATAAACCCAAAAGGGCCGAAGGCTG |
| rDNA-down-BPC-F | CCTTCGGCCCTTTTGGGTTTATTAGTGACGCGCATGAATGG |
| Ku70-up-R | TGGAACAGCCCCACATCGTCATCGTTCTCCAGA |
| Ku70-hisG-F | ACGATGACGATGTGGGGCTGTTCCAGTCAATCAG |
| BPC-R | GAAGTAGCCAGAGAAATGTCC |
| BPC-F | GCTGGAACTTGGGACATTTCT |
| Ku70-mig1t-R | ACGCCGGTGTAAAAACCCAAAAGGGCCGAAGGCTG |
| Ku70-down-BRC-F | CCCTTTTGGGTTTTTACACCGGCGTTATGCTGTT |
| hisG-GPD2p-PK-R | GGACATCCTACTGCGCGGATCTTCCAGTGGTGCA |
| GPD2p-PK-F | CCACTGGAAGATCCGCGCAGTAGGATGTCCTGCACG |
| GPD2p-B. b PK-R | CACGGGGCTAGTCATTGTTGATGTGTGTTTAATTCA |
| B.b PK-F | AAACACACATCAACAATGACTAGCCCCGTGATCGGC |
| B.b PK-R | ATCGACCGGCCAGTGTTACTCGTTGTCGCCGGCGGT |
| mig1t-B. b PK-F | GGCGACAACGAGTAACACTGGCCGGTCGATAATTTA |
| mig1t-TEF1p-R | GCCAACCCGGTCTCTAAACCCAAAAGGGCCGAAGGC |
| TEF1p-mig1t-F | GGCCCTTTTGGGTTTAGAGACCGGGTTGGCGGCGTA |
| TEF1p-B. s PTA-R | CAGGTCGGCAACCATTTTGAATGATTCTTATACTCA |
| B. s PTA-F | TAAGAATCATTCAAAATGGTTGCCGACCTGTTCTCC |
| B. s PTA-R | CCCACGTTGCCGGTCTTACAGAGCCTGAGCGGCGGTA |
| xpr2t-B. s PTA-F | GCTCAGGCTCTGTAAGACCGGCAACGTGGGGACAGG |
| xpr2t-rDNAd-PP-R | CGAGGCATTTGGCTATCGGACACGGGCATCTCACT |
| rDNA-down-PP-F | GATGCCCGTGTCCGATAGCCAAATGCCTCGTCATCTA |
| TEF1p-C. k PTA-R | TTTGAATGATTCTTATACTCACATCAGTTTAACCAT |
| C. k PTA-F | TAAGAATCATTCAAAATGGTTAAACTGATGGAGAAC |
| C. k PTA-R | CCCACGTTGCCGGTCTTAACCCTGAGCCTGAGCCT |
| xpr2t-C. k PTA-F | CAGGCTCAGGGTTAAGACCGGCAACGTGGGGACAGG |
| GPD2p-L. m PK-R | GTCAAAGTCGGCCATTGTTGATGTGTGTTTAATTCA |
| L. m PK-F | AAACACACATCAACAATGGCCGACTTTGACTCTAA |
| L. m PK-R | ATCGACCGGCCAGTGTTATTTGAGGGGGGACCAGGTC |
| mig1t-L. m PK-F | TCCCCCCTCAAATAACACTGGCCGGTCGATAATTTA |
| pUC19-XPR2d-F | GATGCCCGTGTCCGATTTCGGAGGCGATCTGGTCA |
| pUC19-XPR2u-R | GACTGGAACAGCCCCTTCCTGTCTACACGGATGGA |
| XPR2u-hisG-F | CCGTGTAGACAGGAAGGGGCTGTTCCAGTCAATCA |
| PK-mig1t-R | GATGGATGAGATAGCTGGGGAAA |
| PK-mig1t-F | ACTCACCTGTTTTCCCCAGC |
| PTA-xpr2t-XPR2-R | CAGATCGCCTCCGAAATCGGACACGGGCATCTCACTT |
| pUC19-pox4d-F | GATGCCCGTGTCCGAGTATCTCAGGGCAATGGTGAG |
| pUC19-pox4u-R | GACTGGAACAGCCCCCGAAACCCGACTAATTGACTA |
| Pox4u-hisG-F | ATTAGTCGGGTTTCGGGGGCTGTTCCAGTCAATCA |
| PTA-xpr2t-pox4-R | ATTGCCCTGAGATACTCGGACACGGGCATCTCACTT |
| pUC19-Leu2-POX4-up-R | GCGACGACGGAATTCCGAAACCCGACTAATTGACTA |
| POX4-Leu2-F | ATTAGTCGGGTTTCGGAATTCCGTCGTCGCCTGAGT |
| POX4-Leu2-R | ATTGCCCTGAGATACGAATTCATGTCACACAAACCG |
| pUC19-Leu2-POX4-down-F | GTGTGACATGAATTCGTATCTCAGGGCAATGGTGAG |

**Table S3.** Primers used in this study for colony PCR.

| Name | Sequence (5` to 3`) |
| --- | --- |
| gRNA-CP-F | TGCAGCTGGCACGACAGGTTT |
| gRNA-ku70-R | CCAAGGCCTT ATGAAGAGTT |
| gRNA-ku80-R | GAAGGTTGTTCTGGCTAGGA |
| gRNA-rDNA-R | TAAGAGAGCATAGTTACTCC |
| gRNA-D17-R | GTCGTCACCTATATTACGGA |
| gRNA-lip1-R | TCCATTCCTGGTTGCCGAGC |
| gRNA-pox3-R | ATTAGCTACCGGTACAAGGG |
| gRNA-pox4-R | ACGACAGTTCTCGTCAACTC |
| gRNA-pox5-R | GGATAGGGTGAAGTCAGAGG |
| gRNA-xpr2-R | CGTCGACCAGAGAGTCCAGC |
| CarB-CP-F | CGGACGATTCCTGGACTTCAT |
| CarB-CP-R | AGTAGAAGGAAATAGAAGACG |
| CarRP-CP-F | ATCCTGTGGTACGCTTGTCCT |
| CarRP-CP-R | CAGGATGTCCCAAGACACGGA |
| CCD1-CP-F | ACACCTGCCCGAGTGTCTGAA |
| CCD1-CP-R | CGTAGCCGAAGGTGAACATCT |
| Ura3-CP-F | TCTGGTCGAGGTAGCGTTTGA |
| Ura3-CP-R | TGGGTCTTGACGACAAGGGAG |
| idit-CP-F | AGTAGGGATTGGCGAAGTAAT |
| ERG20p-CP-R | CTTTCCACCCACACAGTTGTA |
| erg10t-CP-F | GAGAAAGGGGTGCTTGGAGAT |
| ERG13p-CP-R | CAATTCGAGAAGGCACGTAGA |
| ERG8t-CP-F | TGTCCACCGCGTACAAATGTT |
| ERG12p-CP-R | AGGAGGAACGTAATGCAGAAC |
| ERG12t-CP-F | CGCCCGTTACAACTAGCTTTA |
| ERG19p-CP-R | ACCCCCAACTATCATGTTGCT |
| TEF1p-CP-F | GGTTGGGACTTTAGCCAAGGG |
| EXP1p-CP-F | GCCCATCAGGCAATCCAATTAAG |
| GPD2p-CP-F | TGAGGCACGCCCTCGAATTTG |
| ERG10-CP-R | GCAACATGGCCATTGGGATCAG |
| ERG13-CP-R | GTTGACGTTGTCCACACCCTC |
| ERG12-CP-R | TCCAGAAGCGCCAAGCTAGACA |
| ERG8-CP-R | TGCGTGCTGTCAATTTGCGAGT |
| ERG19-CP-R | GTTGTTCTCGGACACGATCTTG |
| ERG20-CP-R | GAGTGCTTGTCCAGAGAGAACC |
| IDI-CP-R | GGTCCACATGTTGGCAAAGGTG |
| GGS1-CP-R | ACCCTGACCTCTATGCAAGTTG |
| tHMG1-CP-R | GACTGCTGGGAGATGATAGATC |
| B.B PK-CP-R | CTCGTCCTTGGTGATCTTGGG |
| B.s PTA-CP-R | CTCCTTGGTCTTGATGATCTGCA |
| ku70-β-CP-F | CACGTGTTCTCCACGGCATTC |
| TEF1p-CP-R | CCGGGGGAGGTTTGATATGTG |

1. Zhang C, Chen X, Lindley ND, Too HP. A "plug-n-play" modular metabolic system for the production of apocarotenoids. Biotechnol Bioeng. 2018;115(1):174-183.
2. Werner N, Ramirez-Sarmiento CA, Agosin E. Protein engineering of carotenoid cleavage dioxygenases to optimize β-ionone biosynthesis in yeast cell factories. Food Chem. 2019;299:125089.
3. Czajka JJ, Nathenson JA, Benites VT, Baidoo EEK, Cheng Q, Wang Y, et al. Engineering the oleaginous yeast *Yarrowia lipolytica* to produce the aroma compound beta-ionone. Microb Cell Fact. 2018;17(1):136.
