# Supplementary material for: A modular pathway engineering strategy for the high-level production of β-ionone in Yarrowia lipolytica
Source: Microb Cell Fact. 2020 Feb 27;19:49. doi: 10.1186/s12934-020-01309-0 (PMC7045511; doi:10.1186/s12934-020-01309-0)
Supplement: Supplementary file 3 — Additional file 3. Methods for plasmids construction. [file 12934_2020_1309_MOESM3_ESM.docx]

**Additional file 3**

**Methods for plasmids construction**

**pUC19-HA plasmid:** For the construction of pUC19-ku70-HA, the upstream and downstream homologous arms were amplified from *Y. lipolytica* po1f genomic DNA with the primers ku70-up-NotI-F/ku70-up-R and ku70-down-F/ku70-down-NotI-R, respectively. Then the overlap-extension PCR of the two fragments was performed to generate homologous arm fragment. The pUC19 plasmid was linearized by the amplification with primers pUC19-tong-F/R. The resulted linearized plasmid and homologous arm fragment were then assembled by Gibson assembly method, yielding pUC19-ku70-HA. The other pUC19-HA plasmids were constructed following the same protocol. The Q5® High-Fidelity DNA Polymerase (NEB) was used for all the PCR amplifications. The relative primers are listed in Table S2.

**pCAS1yl-gRNA series**: For the construction of pCAS1yl-ku70, two fragments pCAS1yl-ku70-f1 and pCAS1yl-ku70-f2 were obtained by the amplification from pCAS1yl with primers pCAS1yl-F/ku70-gRNA-R and ku70-gRNA-F/pCAS1yl-R, respectively. The resulted fragments were then assembled to generate pCAS1yl-ku70 by Gibson assembly method. All the other pCAS1yl-gRNA plasmids were constructed following the same protocol. The relative primers are listed in Table S2.

**pUC19-P_TEF1_-CarB-xpr2t/pUC19-P_EXP1_-CarRP-lip2t/pUC19-P_GPD2_-CCD1-mig1t**: The promoters (*TEF1p, EXP1p* and *GPD2p*) and the terminators (*xpr2t*, *lip2t* and *mig1t*) were obtained by the amplification from the genomic DNA of *Y. lipolytica* po1f with primers: TEF1p-F/R, EXP1p-F/R, GPD2p-F/R, xpr2t-F/R, lip2t-F/R and mig1t-F/R, respectively. The *CarB*, *CarRP* and *CCD1* genes were amplified from the synthesized templates with primers CarB-F/R, CarRP-F/R and CCD1-F/R, respectively. Then the promoter, gene and terminator were assembled to generate three plasmids by Gibson assembly method in the following order: P_TEF1_-CarB-xpr2t, P_EXP1_-CarRP-lip2t, P_GPD2_-CCD1-mig1t.

**pUC19-rDNAup-HUH-CarB-CarRP-CCD1-rDNAdown**: The HisG-Ura3-HisG fragment was divided into two parts by the amplification from pTA-HUH with primers rDNA-hisG-F/Ura3-R and Ura3-F/rDNA-hisG-R, respectively. CarB expression cassette was amplified from pUC19-P_TEF1_-CarB-xpr2t with primers TEF1p-hisG-F/xpr2t-EXP1p-R. CarRP expressed cassette was amplified from pUC19-P_EXP1_-CarRP-lip2t with primers EXP1p-xpr2t-F/lip2t-GPD2p-R. CCD1 expression cassette was amplified from pUC19-P_GPD2_-CCD1-mig1t with primers GPD2p-lip2t-F/mig1t-rDNA-R. The plasmid pUC19-NotI-rDNAup-rDNAdown-NotI was linearized by the amplification with primers rDNA-down-BPC-F/rDNAu-R. All the six fragments were then assembled to yield pUC19-NotI-rDNAup-HUH-CarB-CarRP-CCD1-rDNAdown-NotI by Gibson assembly method.

**pUC19-ku70up-HUH-CarB-CarRP-CCD1-ku70down**: The homologous arm fragment ku70down-pUC19-ku70up was amplified from pUC19-ku70-HA with primers ku70-down-BPC-F/ku70-up-R. Three fragments were obtained by the amplification from pUC19-NotI-rDNAup-HUH-CarB-CarRP-CCD1-rDNAdown-NotI with primers ku70up-hisG-F/Ura3-R, Ura3-F/BPC-R and BPC-F/ku70-mig1t-R, respectively. The four resulted fragments were then assembled to generate pUC19-ku70up-HUH-CarB-CarRP-CCD1-ku70down by Gibson assembly method.

**pUC19-ku80up-HUH-P_TEF1_-GGS1-xpr2t-ku80down:** *GGS1* was amplified from the genomic DNA with primers GGS1-P-F/GGS1-t-R. xpr2t-pUC19-TEF1p was amplified from pUC19-P_TEF1_-CarB-xpr2t with primers pUC19-GGS1-F/R. The two resulted fragments were assembled to generate pUC19**-**P_TEF1_-GGS1-xpr2t by Gibson assembly method. Primers Ku80-down-F/ku80-up-R were used to amplify the homologous arm fragment from pUC19-ku80-HA, yielding ku80down-pUC19-ku80up. The HUH fragment was divided into two parts by the amplification from pTA-HUH with primers ku80-hisG-F/Ura3-R and Ura3-F/ku80-hisG-R, respectively. GGS1 expression cassette was obtained from pUC19**-**P_TEF1_-GGS1-xpr2t by the amplification with primers TEF1p-GGS1-F/xpr2t-GGS1-R. The four resulted fragments were then assembled to generate pUC19-ku80up-HUH-P_TEF1_-GGS1-xpr2t-ku80down by Gibson assembly method.

**pUC19-D17up-HUH-P_EXP1_-tHMG1-lip2t-D17down:** *tHMG1* was amplified from the genomic DNA with primers tHMG1-P-F/tHMG1-t-R. lip2t-pUC19-EXP1p was amplified from pUC19-P_EXP1_-CarRP-lip2t with primers pUC19-tHMG1-F/R. The two resulted fragments were assembled to generate pUC19-P_EXP1_-tHMG1-lip2t by Gibson assembly method. Primers D17-down-F/D17-up-R were used to amplify the homologous arm fragment from pUC19-D17-HA, yielding D17-down-pUC19-D17-up. The HUH fragment was divided into two parts by the amplification from pTA-HUH with primers D17-hisG-F/Ura3-R and Ura3-F/D17-hisG-R, respectively. tHMG1 expression cassette was obtained by the amplification from pUC19**-**P_EXP1_-tHMG1-lip2t with primers EXP1p-tHMG1-F/lip2t-tHMG1-R. The four resulted fragments were then assembled to generate pUC19-D17-HUH-P_EXP1_-tHMG1-lip2t-D17 by Gibson assembly method.

**pUC19-lip1up-P_TEF1_-ERG10-erg10t-P_GPD2_-ERG13-erg13t-HUH-lip1down:** ERG10-erg10t and ERG13-erg13t were amplified from the genomic DNA with primers ERG10-F/R and ERG13-F/R, respectively. pUC19-TEF1p was obtained by the amplification from pUC19-P_TEF1_-CarB-xpr2t with primers pUC19-tong-F/pUC19-TEF1p-R. pUC19-GPD2p was obtained by the amplification from pUC19-P_GPD2_-CCD1-mig1t with primers pUC19-tong-F/pUC19-GPD2p-R. The resulted fragments were assembled by Gibson assembly method to generate pUC19-P_TEF1_-ERG10-erg10t and pUC19-P_GPD2_-ERG13-erg13t, respectively. Primers Lip1-down-F/lip1-up-R were used to amplify the homologous arm fragment from pUC19-lip1-HA, yielding lip1-down-pUC19-lip1-up. The HUH fragment was divided into two parts by the amplification from pTA-HUH with primers lip1-hisG-F/Ura3-R and Ura3-F/lip1-hisG-R, respectively. ERG10 and ERG13 expression cassettes were obtained from the constructed plasmids by the amplification with primers ERG10-TEF1p-F/erg10t-ERG13-R and ERG13-ERG10-F/erg13t-R, respectively. The five resulted fragments were then assembled to generate pUC19-lip1up-P_TEF1_-ERG10-erg10t-P_GPD2_-ERG13-erg13t-HUH-lip1down by Gibson assembly method.

**pUC19-pox3up-P_EXP1_-IDI-idit-P_TEF1_-ERG20-erg20t-HUH-pox3down:** ERG20-erg20t and IDI-idit were amplified from the genomic DNA with primers ERG20-F/R and IDI-F/R, respectively. pUC19-TEF1p was obtained by the amplification from pUC19-P_TEF1_-CarB-xpr2t with primers pUC19-tong-F/pUC19-TEF1p-R. pUC19-EXP1p was obtained by the amplification from pUC19-P_EXP1_-CarRP-lip2t with primers pUC19-tong-F/pUC19-EXP1p-R. The resulted fragments were assembled by Gibson assembly method to generate pUC19-P_TEF1_-ERG20-erg20t and pUC19-P_EXP1_-IDI-idit, respectively. Primers Pox3-down-F/pox3-up-R were used to amplify the homologous arm fragment from pUC19-pox3-HA to yield pox3-down-pUC19-pox3-up. The HUH fragment was divided into two parts by the amplification from pTA-HUH with primers pox3-hisG-F/Ura3-R and Ura3-F/pox3-hisG-R, respectively. IDI and ERG20 expression cassettes were obtained from the constructed plasmids by the amplification with primers IDI-EXP1p-F/idit-ERG20-R and ERG20-IDI-F/erg20t-R, respectively. The five resulted fragments were then assembled to generate pUC19-pox3up-P_EXP1_-IDI-idit-P_TEF1_-ERG20-erg20t-HUH-pox3down by Gibson assembly method.

**pUC19-pox5up-P_EXP1_-ERG8-erg8t-P_TEF1_-ERG12-erg12t-P_GPD2_-ERG19-erg19t-HUH-pox5down:** ERG8-erg8t, ERG12-erg12t and ERG19-erg19t were amplified from the genomic DNA with primers ERG8-F/R, ERG12-F/R and ERG19-F/R, respectively. pUC19-EXP1p was obtained by the amplification from pUC19-P_EXP1_-CarRP-lip2t with primers pUC19-tong-F/pUC19-EXP1p-R. pUC19-TEF1p was obtained by the amplification from pUC19-P_TEF1_-CarB-xpr2t with primers pUC19-tong-F/pUC19-TEF1p-R. pUC19-GPD2p was obtained by the amplification from pUC19-P_GPD2_-CCD1-mig1t with primers pUC19-tong-F/pUC19-GPD2p-R. The resulted fragments were assembled by Gibson assembly method to generate pUC19-P_EXP1_-ERG8-erg8t， pUC19-P_TEF1_-ERG12-erg12t and pUC19-P_GPD2_-ERG19-erg19t, respectively. Primers Pox5-down-F/pox5-up-R were used to amplify the homologous arm fragment from pUC19-pox5-HA, yielding pox5-down-pUC19-pox5-up. The HUH fragment was divided into two parts by the amplification from pTA-HUH with primers pox5-hisG-F/Ura3-R and Ura3-F/pox5-hisG-R, respectively. ERG8, ERG12 and ERG19 expression cassettes were obtained from the constructed plasmids by the amplification with primers ERG8-EXP1p-F/erg8t-ERG12-R, ERG12-ERG8-F/erg12t-ERG19-R and ERG19-ERG12-F/erg19t-R, respectively. The resulted fragments were then assembled to generate pUC19-pox5up-P_EXP1_-ERG8-erg8t-P_TEF1_-ERG12-erg12t-P_GPD2_-ERG19-erg19t-HUH-pox5down by Gibson assembly method.

**pUC19-P_TEF1_-PK-xpr2t/pUC19-P_GPD2_-PTA-mig1t:** Two PTA genes were amplified from the synthesized templates with primers B. s PTA-F/R and C. k PTA-F/R, respectively. Primers xpr2t-PTA-F/TEF1p-PTA-R were used to amplify xpr2t-pUC19-TEF1p from pUC19-P_TEF1_-CarB-xpr2t. The resulted fragments were assembled by Gibson assembly method to generate pUC19-P_TEF1_-B. s PTA-xpr2t and pUC19-P_TEF1_-C. k PTA-xpr2t, respectively. The PK genes were amplified from the synthesized templates with primers B. b PK-F/R and L. m-PK-F/R, respectively. Primers mig1t-PK-F/GPD2p-PK-R were used for the amplification of mig1t-pUC19-GPD2p from pUC19-P_GPD2_-CCD1-mig1t. The resulted fragments were assembled by Gibson assembly method to generate pUC19-P_GPD2_-B. b PK-mig1t and pUC19-P_GPD2_-L. m PK-mig1t, respectively.

**pUC19-rDNAup-HUH-P_TEF1_-PK-xpr2t-P_GPD2_-PTA-mig1t-rDNAdown:** The PK and PTA expression cassettes were obtained by the amplification from pUC19-P_TEF1_-PTA-xpr2t and pUC19-P_GPD2_-PK-mig1t with primers GPD2p-PK-F/mig1t-TEF1p-R and TEF1p-mig1t-PTA-F/xpr2t-rDNAd-PP-R, respectively. The HUH fragment was divided into two parts by the amplification from pTA-HUH with primers rDNA-hisG-F/Ura3-R and Ura3-F/hisG-GPD2p-PK-R, respectively. The pUC19-NotI-rDNAup-rDNAdown-NotI plasmid was linearized by the amplification with primers rDNA-down-PP-F/rDNAu-R. The five resulted fragments were then assembled to yield pUC19-rDNAup-HUH-P_TEF1_-PK-xpr2t-P_GPD2_-PTA-mig1t-rDNAdown by Gibson assembly method.

**pUC19-XPR2up-HUH-P_TEF1_-B. b PK-xpr2t-P_GPD2_-B. c PTA-mig1t-XPR2down:** The HUH-B. b PK-B. c PTA fragment was divided into three parts by the amplification with primers xpr2u-hisG-F/Ura3-R, Ura3-F/PK-mig1t-R and PK-mig1t-F/PTA-xpr2t-XPR2-R, respectively. The pUC19-NotI-XPR2up-XPR2down-NotI plasmid was linearized by the amplification with primers pUC19-XPR2d-F/pUC19-XPR2u-R. The five resulted fragments were then assembled to yield pUC19-XPR2up-HUH-P_TEF1_-PK-xpr2t-P_GPD2_-PTA-mig1t-XPR2down by Gibson assembly method.

**pUC19-pox4up-HUH-P_TEF1_-B. b PK-xpr2t-P_GPD2_-B. c PTA-mig1t-pox4down:** The HUH-B. b PK-B. c PTA was divided into three parts by the amplification with primers pox4u-hisG-F/Ura3-R, Ura3-F/PK-mig1t-R and PK-mig1t-F/PTA-xpr2t-pox4-R, respectively. The pUC19-NotI-pox4up-pox4down-NotI plasmid was linearized by the amplification with primers pUC19-pox4d-F/pUC19-pox4u-R. The five resulted fragments were then assembled to yield pUC19-POX4up-HUH-P_TEF1_-PK-xpr2t-P_GPD2_-PTA-mig1t-POX4down by Gibson assembly method.

**pUC19-pox4up-Leu2-pox4down:** Leu2 expressed cassette was amplified from pINA1269 with primers POX4-Leu2-F/R. The pUC19-NotI-POX4up-POX4down-NotI plasmid was linearized by the amplification with primers pUC19-LEU2-POX4-down-F/pUC19-LEU2-POX4-up-R. The two fragments were then assembled to yield pUC19-POX4up-Leu2-POX4down by Gibson assembly method.

All plasmids containing expression cassettes were sequence-confirmed before transformation into *Y. lipolytica*.
